# ZYP1 is required for obligate cross-over formation and cross-over interference in *Arabidopsis*

**DOI:** 10.1073/pnas.2021671118

**Published:** 2021-03-29

**Authors:** Martin G. France, Janina Enderle, Sarah Röhrig, Holger Puchta, F. Chris H. Franklin, James D. Higgins

**Affiliations:** ^a^Department of Genetics and Genome Biology, University of Leicester, LE1 7RH Leicester, United Kingdom;; ^b^Botanical Institute, Molecular Biology and Biochemistry, Karlsruhe Institute of Technology, Karlsruhe 76131, Germany;; ^c^School of Biosciences, University of Birmingham, B15 2TT Birmingham, United Kingdom

**Keywords:** meiosis, recombination, synaptonemal complex, cross-over interference

## Abstract

The synaptonemal complex is a tripartite proteinaceous ultrastructure that forms between homologous chromosomes during prophase I of meiosis in the majority of eukaryotes. It is characterized by the coordinated installation of transverse filament proteins between two lateral elements and is required for wild-type levels of crossing over and meiotic progression. We have generated null mutants of the duplicated *Arabidopsis* transverse filament genes *zyp1a* and *zyp1b* using a combination of T-DNA insertional mutants and targeted CRISPR/Cas mutagenesis. Cytological and genetic analysis of the *zyp1* null mutants reveals loss of the obligate chiasma, an increase in recombination map length by 1.3- to 1.7-fold and a virtual absence of cross-over (CO) interference, determined by a significant increase in the number of double COs. At diplotene, the numbers of HEI10 foci, a marker for Class I interference-sensitive COs, are twofold greater in the *zyp1* mutant compared to wild type. The increase in recombination in *zyp1* does not appear to be due to the Class II interference-insensitive COs as chiasmata were reduced by ∼52% in *msh5/zyp1* compared to *msh5*. These data suggest that ZYP1 limits the formation of closely spaced Class I COs in *Arabidopsis*. Our data indicate that installation of ZYP1 occurs at ASY1-labeled axial bridges and that loss of the protein disrupts progressive coalignment of the chromosome axes.

## Significance

The synaptonemal complex (SC) is a meiosis-specific proteinaceous ultrastructure required to ensure cross-over (CO) formation in the majority of sexually reproducing eukaryotes. It is composed of two lateral elements adjoined by transverse filaments. Even though the general structure of the SC is conserved throughout kingdoms, phenotypic differences between mutants perpetuate the enigmatic role of the SC. Here, we have used genetic and cytogenetic approaches to show that the transverse filament protein, ZYP1, acts on multiple pathways of meiotic recombination in *Arabidopsis*. ZYP1 is required for CO assurance, thus ensuring that every chromosome pair receives at least one CO. ZYP1 limits the number of COs and mediates CO interference, the phenomenon that reduces the probability of multiple COs forming close together.

The synaptonemal complex (SC) is a proteinaceous ultrastructure that forms between homologous chromosomes (homologs) during midprophase I of meiosis and plays a critical role in coordinating the repair of programmed DNA double-strand breaks (DSBs) to form cross-over (CO) products (1, 2). At the onset of leptotene, the sister chromatids are organized into linear looped chromatin arrays conjoined at the loop bases by a protein axis that runs along the chromosomes (3, 4). Early steps in the recombination pathway enable the loose alignment of homolog axes at a distance of ∼400 nm (5). Formation of the SC then initiates and continues throughout zygotene via progressive installation of transverse filaments (TFs) that run perpendicular to the aligned homolog axes (referred to as lateral elements in the context of the SC), ultimately bringing them into close apposition along their entire length at a distance of ∼100 nm (2, 5). Installation of the TFs starts at multiple synapsis initiation sites that correspond to future Class I COs in *Saccharomyces cerevisiae* (6). In species with larger chromosomes such as S*ordaria macrospora*, synapsis initiates from CO-designated sites as well as additional sites whose distribution also appears sensitive to interference (1, 5). In *Arabidopsis thaliana*, 20 to 25 synapsis initiation sites per cell indicate a ∼2- to 2.5-fold excess over COs and in barley 76 synapsis initiation sites, versus 17 chiasmata reveal a ∼4.5-fold excess (7, 8). Full synapsis denotes the onset of pachytene and is maintained throughout this stage during which time CO formation is completed. As prophase I progresses to diplotene/diakinesis, the SC is disassembled.

TFs have been described in a variety of organisms, and in most cases, they are composed of a single protein. These include Zip1 in budding yeast, C(3)G in *Drosophila melanogaster*, SYCP1 in mouse, ZYP1 in *A. thaliana* (encoded by duplicated genes, *ZYP1a* and *ZYP1b*), ZEP1 in rice (*Oryza sativa*), and ZYP1 in barley (*Hordeum vulgare*) (9–16). *Caenorhabditis elegans* is an exception that possesses six TF proteins (SYP1-6) required for normal synapsis (17–22). Despite a striking lack of homology between the TFs at the primary amino acid sequence level, they share very similar structures, comprising a globular N-terminal domain linked to another globular domain at the C terminus via a long alpha helical central region that is able to form large stretches of parallel, in-register, homodimeric coiled coils (23). Studies have shown that the TFs are oriented such that the C termini are associated with lateral elements potentially interacting with DNA, while the N-terminal domains localize to the central region (2, 24). Evidence suggests that the overall three-dimensional macromolecular organization of the SC is also somewhat conserved. Analyses in mouse, *Drosophila*, and *H. vulgare* (barley) strongly suggest that these organisms form SCs with a bilayer of TFs (25–28). A multilayered structure is also supported by studies in *Blabs cribrosa* (beetle) (29, 30). However, key aspects of the organization of the TFs within the SC remain a matter of debate. Initially, analysis of *zip1* mutants in *S. cerevisiae* suggested that the TFs comprise a tetramer of two opposing Zip1 dimers with their *N* termini forming overlapping interactions in the central region of the SC (31). X-ray crystallographic studies of the human TF, SYCP1, report that the protein forms a tetrameric building block that self-assembles into a zipper-like lattice through “head-to-head” N-terminal interactions in the SC central region and “back-to-back” interactions between adjacent C-terminal dimers at the lateral elements (24). In contrast, analysis of the mouse SC using electron tomography has led to the proposal that the SC has a more dynamic structure with TF dimers forming a variety of less regimented interactions as part of an irregular single plane. However, this model appears inconsistent with other studies in mouse which support a more ordered structure (25, 26).

Mutant analysis has demonstrated that TF proteins are essential for assembly of the SC central region and thus homolog synapsis. These also confirm an important role in the control of CO formation but with some variation between organisms. Studies of *zip1* mutants in *S. cerevisiae* have shown that the Zip1 protein is a member of the ZMM group of proteins comprising Zip1, Zip2, Zip3, Zip4, Msh4, Msh5, and Mer3 that are required for the formation of Class I interfering COs (32). CO interference is a patterning mechanism that ensures even spacing of COs along the chromosomes (33–35). In *S. cerevisiae* and *Arabidopsis*, Class I COs account for ∼85% of total COs and the remaining Class II COs (∼15%) are randomly distributed (36–38). However, in plants, Zip1 orthologs appear to be functionally independent of the other ZMM proteins for CO formation (14–16). Genetic analysis of *S. cerevisiae zip1* deletion mutants revealed a modest reduction in CO formation ∼30 to 40% with residual COs no longer exhibiting CO interference leading to the suggestion that the SC may mediate this process (10). Subsequent studies based on a molecular analysis of recombination intermediates in *zip1* and other *zmm* mutants argue against a role for the SC in mediating interference as they indicate that the fate of DSBs is designated at an early stage in the recombination pathway prior to installation of the SC (32, 39). In female *Drosophila* lacking the TF protein C(3)G, DSB formation is thought to be reduced and they fail to form COs, although SC formation is independent of recombination (12). These authors also report that analysis of flies expressing a mutant version of the protein reveals that a complete SC is not required for CO interference (12). A major reduction in COs of ∼90% is also observed in mouse *sycp1* mutants although DSB formation appears normal (13). Similarly in *C. elegans* (in which SC installation occurs at pairing centers), *syp-1* and *syp-2* null mutants recombination is initiated but COs do not form (17, 18). A further study in which the SC central region was partially depleted by RNA interference (RNAi)–induced *SYP-1* knockdown found that CO interference was reduced leading to an increase in COs, suggesting a role for the SC in limiting COs (40).

TFs have been studied in several plant species including *Arabidopsis*, barley, and rice (14–16). Analysis of *Tos17* insertion mutants of the rice TF gene *ZEP1* demonstrated that in common with other organisms, it is essential for SC formation and affects CO formation (16). However, rather than displaying a reduction in COs, analysis of the short arm of chromosome 11 revealed a more than threefold increase in COs in *zep1* mutants (16). Like rice, barley is a member of the grass family (Poaceae), and in common with rice, RNAi knockdown lines of the TF protein HvZYP1 are defective in SC formation, but in contrast, CO formation is reduced to ∼25% of wild-type levels (15). In *Arabidopsis*, the TF protein, ZYP1, is encoded by functionally redundant duplicated genes, *ZYP1a* and *ZYP1b*, which share 93% homology and are encoded within 2 kb of each other on opposite strands of chromosome 1. Individual *zyp1a* and *zyp1b* mutants are fertile and possess only mild meiotic phenotypes, and as isolation of a double mutant has thus far proved intractable, functional analysis of ZYP1 has relied on RNAi knockdown lines (14). As expected, these lines failed to assemble an SC. Chiasma frequency was reduced by ∼20 to 30% and based on metaphase I bivalent shapes, they appeared to exhibit interference, but a proportion involved ectopic recombination with nonhomologs (14).

Although existing studies imply that there may be some variation in the role of the SC in relation to CO control in plants, the studies in *Arabidopsis* and barley were based on RNAi knockdown lines rather than TF mutants. Hence to address this issue, we have generated CRISPR/Cas *zyp1a/zyp1b* mutants. This has enabled a detailed analysis of ZYP1 function in *Arabidopsis*, revealing that it is required for formation of the obligate CO and implementation of CO patterning. Loss of the protein also disrupts the normal program of homolog coalignment during prophase I.

## Materials and Methods

### Plant Materials.

*zyp1a-1* (SALK_040213) (14); *zyp1a-2* (CRISPR/Cas9 derived 14 bp deletion in exon 3 c.298_311 del GATGAGAAGCTTTG); *zyp1a-3* (CRISPR/Cas9 derived 11 bp deletion in exon 3 c.301_311 del GAGAAGCTTTG); *zyp1b-1* (SALK_050581) (14); *zyp1b-1* (CRISPR/Cas9 derived 1 bp insertion in exon 1 c.60_61 ins T); *asy1-4* (SALK_046272) (41); *asy3-1* (SALK_143676) (42); *pch2-1* (SAIL_1187_C06) (43); *mlh3-1* (SALK_015849) (44); and *msh5-1* (SALK_110240) (45) were acquired from Nottingham Arabidopsis Stock Centre. For obtaining *zyp1* double mutants, CRISPR/Cas9 mediated mutagenesis was performed (46). For targeting *ZYP1A* and *ZYP1B*, the protospacers 5′ GAA​GAT​GAG​AAG​CTT​TGG​AG 3′ and 5′ GGA​TCG​GCG​AAG​ACG​TAC​TT 3′ were cloned, respectively, into pEn-C1.1 and integrated into the expression vector pDe-CAS9 by gateway cloning. FTLs are as follows: 420 (Chr 3: transfer DNA [T-DNA]-1: GFP-256,516; TDNA-2: dsRed2-5,361,637) (47), I3bc (Chr 3: TDNA-1 CFP 498,916; TDNA-2 YFP 3,126,994, TDNA-3 dsRed2-4,319,513) *qrt1-2* (CS8846) (48), and I5ab (Chr 5: TDNA-1 dsRed2 18,164,269; TDNA-2 YFP 23,080,567, TDNA-3 CFP 25,731,311) *qrt1-2* (48). Further information is provided in *SI Appendix*.

### Coimmunofluorescence Analysis of Chromosome Spreads.

Coimmunofluorescence was performed on 4% paraformaldehyde preparations (49) or in 3:1 ethanol:acetic acid fixed material followed by antigen recovery (50). Primary antibodies are the following: rat anti-AtZYP1-C (51), rat/guinea pig anti-AtZYP1-C (1:500) (14), rabbit anti-AtZYP1-N (1:500) (38), guinea pig/rat anti-ASY1 (1:500) (52), rabbit anti-HvHEI10 (1:200) (53), and rat anti-AtSMC3 (1:200). Secondary antibodies at 1:200 are the following: goat anti-rabbit AMCA (Jackson ImmunoResearch), goat anti-guinea pig Alexa Fluor 488 (Abcam), goat anti-rat Alexa Fluor 488 (Invitrogen), and goat anti-rabbit DyLight 594 (Vector laboratories). A Nikon Ni-E fluorescence microscope with a DS-Qi1MC CCD camera and NIS-Elements software was used to capture images. Super-resolution microscopy methodology is provided in *SI Appendix*.

## Results

### *zyp1a/b* CRISPR/Cas Null Mutants.

**Fig. 1.**
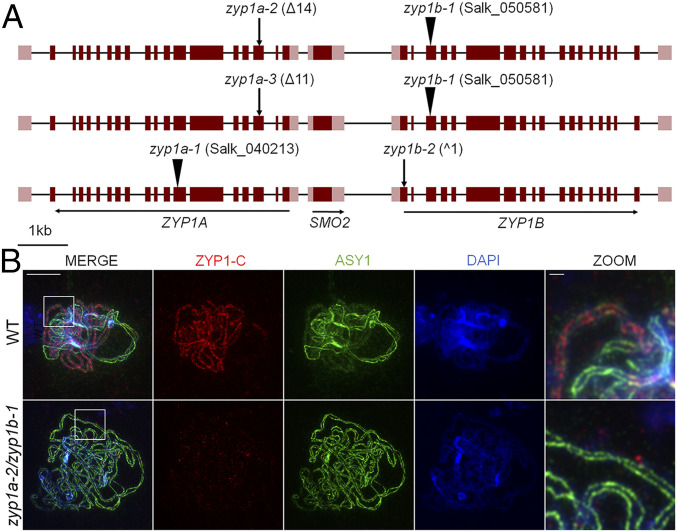
*zyp1* null mutants are asynaptic. (*A*) The ZYP1 locus showing positions of T-DNA insertions and CRISPR/Cas mutations for the three *zyp1* null mutants. Exons are represented by dark red boxes, and noncoding regions of the transcripts are shown in light red. (*B*) Coimmunofluorescence of ASY1 and ZYP1-C terminus on wild-type and *zyp1a-2/zyp1b-1* meiotic prophase I chromosome spread preparations. (Scale bars, 10 µm for main panels and 1 µm for zoom.)

As the duplicated *ZYP1a/b* genes are separated by just 2.1 kb between start codons, the generation of a double knockout line by crossing individual *zyp1a-1* and *zyp1b-1* T-DNA mutants presents a significant challenge, as it would require a rare CO event in the intervening region. Hence, a previous study of ZYP1 function relied on the use of RNAi to downregulate *ZYP1* expression in homozygous *zyp1a-1* and *zyp1b-1* backgrounds (14). Here we have capitalized on the availability of CRISPR/Cas to generate lines in which both *ZYP1* copies are null (Fig. 1 and *SI Appendix*, Fig. S1).

Guide RNAs were designed to target exon 3 in *ZYP1a* and exon 1 in *ZYP1b*, predicted to be included in all known transcript isoforms. Furthermore, as both *ZYP1A* exon 3 and the coding sequence of *ZYP1B* exon 1 are not multiples of three in length (208 and 262 bp, respectively), any aberrant exon skipping would result in a nonsense protein. Three null mutant lines were selected for analysis (*zyp1a-2/zyp1b-1*, *zyp1a-3/zyp1b-1*, and *zyp1a-1/zyp1b-*2) (Fig. 1). To confirm the absence of the ZYP1 protein in the *zyp1* mutants, immunostaining of ZYP1 was performed on prophase I chromosome spreads (14). In a previous study, in wild type, ZYP1 loaded as numerous short stretches between homologs during zygotene that extended until full completion at pachytene (14). Immunolocalization of the chromosome axis protein ASY1 showed a characteristic patterned distribution at zygotene, with bright linear signals in the unsynapsed regions, compared to less intense and more diffuse signals in synapsed regions, having been remodelled by PCH2, a conserved AAA+ ATPase which depletes ASY1 as synapsis progresses (43) (Fig. 1*B*). In contrast, linear ZYP1 staining using N- or C terminus antibodies was never observed in *zyp1* prophase I nuclei (Fig. 1*B* and *SI Appendix*, Fig. S2). Synapsis-dependent depletion of ASY1 was also absent in *zyp1* with the protein localizing as a linear signal of uniform intensity throughout prophase I (Fig. 1*B*). The phenotype observed in *zyp1a-2/zyp1b-1* plants was reproduced in *zyp1a-3/zyp1b-1* and *zyp1a-1/zyp1b-2*, both of which also showed an absence of polymerized ZYP1 and uniform linear ASY1 staining (*SI Appendix*, Fig. S2).

### Chromosome Axes Show Evidence of Presynaptic Coalignment in *zyp1*.

**Fig. 2.**
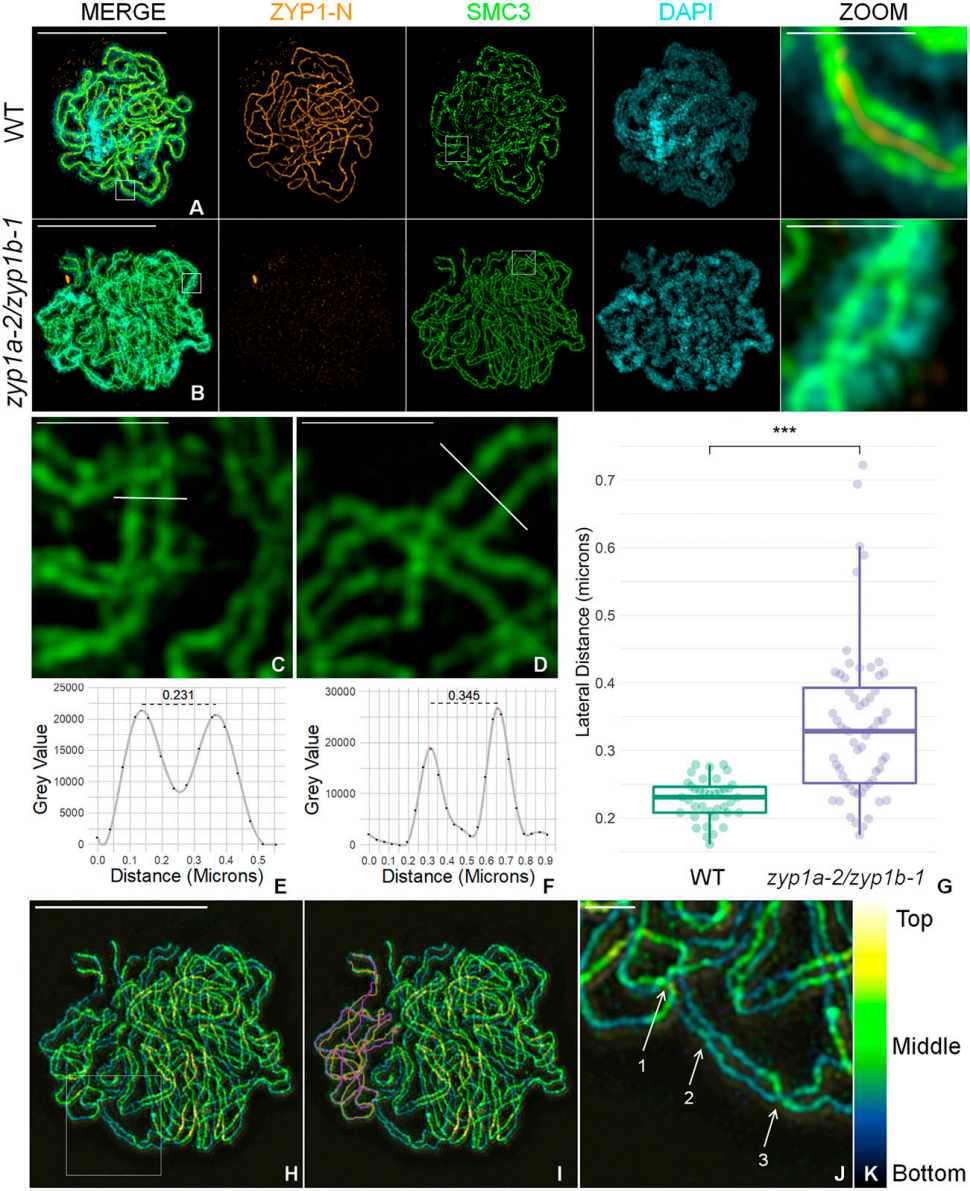
Chromosome axes align at a greater distance in *zyp1*. (*A* and *B*) Coimmunofluorescence of ASY1 and ZYP1-N terminus on wild-type and *zyp1a-2/zyp1b-1* meiotic prophase I chromosome spread preparations (zoom panel is shown in white box in channel merge). (*C* and *D*) Enlarged region (white box from *A* and *B,* SMC3 channel) showing representative lines bisecting the homologous axes used to measure homologous distance. (*E* and *F*) Plot profiles of bisecting lines (from *C* and *D*). (*G*) Comparison of lateral distances between wild type and *zyp1a-2/zyp1b-1.* ***P < 0.001 (Wilcoxon rank sum test). (*H*) False-colored Z-stack projection of a *zyp1* pachytene analog nucleus. (Scale bar, 10 µm.) (*I*) One pair of aligned chromosomes is traced and shown in orange and pink. (*J*) Enlarged region (white box in *A*) shows three features of *zyp1* “pachytene” nuclei (1: axial entanglement, 2: axial bridge, and 3: twisted axes). (*K*) False color encoding for slice depth (z). (Scale bars, *A* [MERGE], *B* [MERGE], *H*: 10 µm; *A* [ZOOM], *B* [ZOOM], *C*, *D*, *J*: 1 µm.)

In wild-type pachytene nuclei, alignment between synapsed lateral elements of the homologs appeared markedly consistent (Fig. 2*A*).

In corresponding *zyp1a-2/zyp1b-1*, nuclei synapsed axes were not observed; nevertheless, they did show evidence of coalignment albeit at a greater distance and with some variability compared to wild type (Fig. 2*B*). To quantify this observation and measure the distance between apposed lateral elements/axes, random rectangular regions of each cell type were sampled, and lines perpendicularly bisecting the two juxtaposed axes were annotated (Fig. 2 *A*–*D*). This revealed that the chromosome axes in the *zyp1a-2/zyp1b-1* mutants were significantly further apart ($\tilde{x}$= 328 nm, Median Absolute Deviation [MAD] = 114 nm, *n* = 60) than that observed for synapsed chromosomes in the wild type ($\tilde{x}$= 231 nm, MAD = 31.1 nm, *n* = 38; Wilcoxon rank sum test W = 354.5, *P* = 1.04 × 10^−8^) (Fig. 2 *E*–*G*). Furthermore, a Fligner–Killeen test confirmed that the distances in the *zyp1* mutant were significantly more variable than the wild type (χ^2^ = 26.47, df = 1, *P* = 2.68 × 10^−7^). Chromosome axial entanglements were also observed in the absence of ZYP1 (Fig. 2 *H*–*K*). These data suggest that presynaptic coalignment occurs in *zyp1a-2/zyp1b-1*, but progression to the closely apposed arrangement observed in wild type cannot in the absence of the ZYP1 protein.

### ZYP1 Loads onto ASY1-Labeled Axial Bridges.

**Fig. 3.**
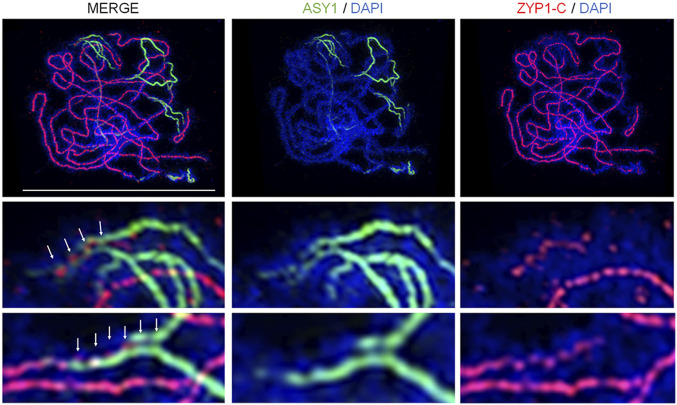
Coimmunofluorescence of ASY1 and ZYP1-C terminus on a late-zygotene wild-type nucleus by structured illumination microscopy. Images show dynamics of axis protein ASY1 unloading and transverse filament protein ZYP1 loading at synaptic forks. Insets and white arrows highlight localization at the axial bridges. (Scale bar, 10 µm.)

We previously showed that the ZYP1 C terminus is embedded in the lateral elements, whereas the N terminus is positioned in the central element using ZYP1-N– and ZYP1-C–labeled antibodies with immunogold and electron microscopy (14), consistent with the canonical model for the majority of SCs in other species (2). TF protein loading is initiated from a patterned set of designated nucleation sites, and then additional TF molecules polymerize until synapsis is complete (54). However, our analysis using super-resolution microscopy on *Arabidopsis* zygotene nuclei suggests that ZYP1 loads intermittently at synaptic forks onto evenly spaced ASY1-labeled interaxis bridges ($\tilde{x}$= 290 nm, SE 18, *n* = 18), previously described in ref. 5. Concomitantly, ZYP1 then “fills in the gaps” between the axial bridges as ASY1 becomes depleted, as opposed to a direct continuous polymerization (Fig. 2). Therefore, ZYP1 may require ASY1 axial bridges for normal loading and adjoining of the lateral elements. In the *zyp1a-2/zyp1b-1* mutant, the persistence of uniform ASY1 staining during prophase I suggests that ASY1 is not depleted from the chromosomes, and axial bridges may persist which could help maintain axis alignment observed in *zyp1a-2/zyp1b-1* (Fig. 3).

### ZYP1 Provides CO Assurance and Maintains Fidelity of Recombination.

**Fig. 4.**
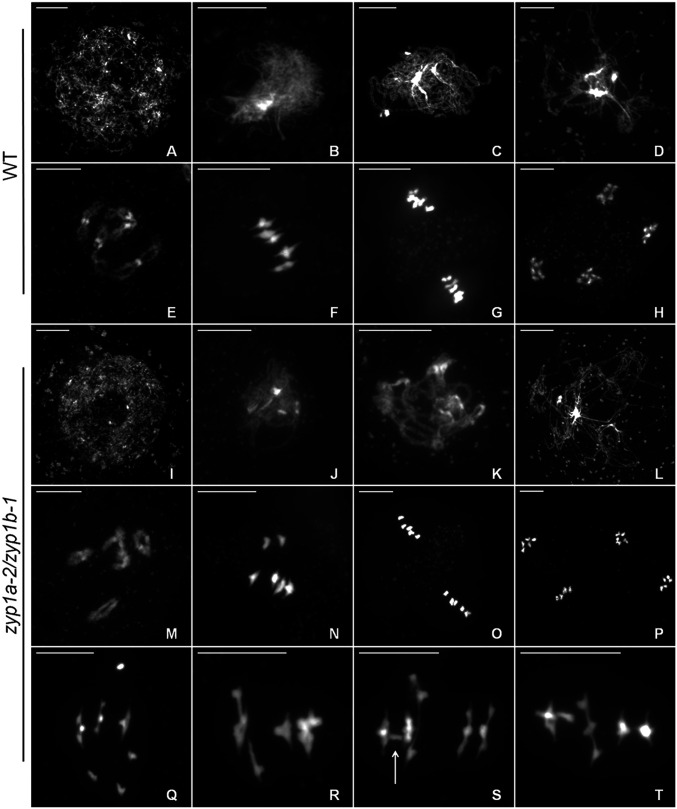
ZYP1 ensures formation of the obligate chiasma. (*A*) Meiotic atlas of DAPI-stained chromosome spreads from wild-type (*A*–*H*) and *zyp1a-2/zyp1b-1* (*I*–*P*) pollen mother cells. (*A* and *I*) Leptotene. (*B* and *J*) Zygotene (and analog). (*C* and *K*) Pachytene (and analog). (*D* and *L*) Diplotene. (*E* and *M*) Diakinesis. (*F* and *N*) Metaphase I. (*G* and *O*) Metaphase II. (*H* and *P*) Telophase II. (*Q*–*T*) Examples of aberrant recombination and possible interlocks in *zyp1a-2/zyp1b-1* at metaphase I. (Scale bars, 10 μm.)

**Fig. 5.**
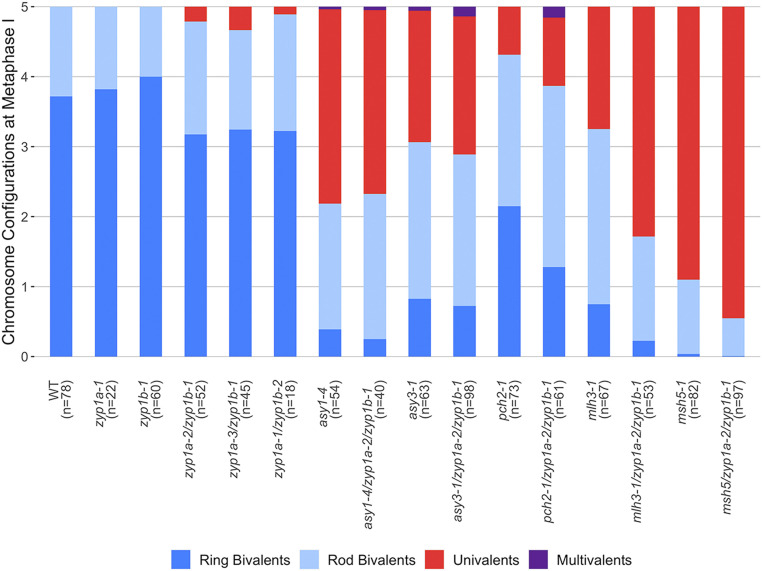
Chromosome configurations at meiotic metaphase I. Chromosome configurations for all genotypes analyzed are presented relative to the five chromosome pairs of *A. thaliana*. The number of cells sampled for each line is shown in brackets.

Metaphase I nuclei in the *zyp1* null mutants exhibited occasional pairs of univalent chromosomes, revealing the loss of the obligate CO and suggesting defective CO assurance. Therefore, a detailed examination of metaphase I nuclei was performed, scoring rods (1 chiasma), rings (>2 chiasmata), and univalents (no chiasmata) for the wild-type, *zyp1a-1*, *zyp1b-1*, *zyp1a-2/zyp1b-1*, *zyp1a-3/zyp1b-1*, and *zyp1a-1/zyp1b-2* mutants. For all three *zyp1* null mutant lines, a small number of univalent chromosomes pairs were observed (*zyp1a-2/zyp1b-1*: 15 pairs from 52 cells; *zyp1a-3/zyp1b-1*: 11 pairs from 45 cells; and *zyp1a-1/zyp1b-2*: 2 pairs from 18 cells), but univalents were never observed in the wild-type (78 cells) nor in the single mutants, *zyp1a-1* (60 cells) and *zyp1b-1* (22 cells) (Figs. 4 and 5).

Chiasmata were also slightly reduced (0.5 to 1) in the *zyp1* null mutants compared to wild type (Table 1). Bonferroni-corrected Wilcoxon rank sum tests confirmed that the reduction in chiasmata between the *zyp1* mutants and the wild type were statistically significant (*zyp1a-2/zyp1b-1*: W = 2789, adjusted *P* value [*P*adj] = 0.000436, *zyp1a-3/zyp1b-1*: W = 2323, *P*adj = 0.00484, and *zyp1a-1/zyp1b-2*: W = 952, *P*adj = 0.0367).

**Table 1.** Chiasma frequency scored at metaphase I for all genotypes

Genotype	*n*	Mean chiasmata ± SD	Minimum chiasmata	Maximum chiasmata	Univalent pairs (proportion)
WT	78	8.92 ± 0.834	7	11	0 (0)
*zyp1a-1*	22	9.00 ± 1.20	6	11	0 (0)
*zyp1b-1*	60	9.13 ± 0.982	6	11	0 (0)
*zyp1a-2/zyp1b-1*	52	8.07 ± 1.55	4	12	15 (0.042)
*zyp1a-3/zyp1b-1*	45	8.12 ± 1.76	2	11	11 (0.066)
*zyp1a-1/zyp1b-2*	18	8.33 ± 1.37	7	12	2 (0.022)
*asy1-4*	54	2.65 ± 1.32	0	6	150 (0.56)
*asy1-4/zyp1a-2/zyp1b-1*	40	2.65 ± 1.31	1	7	105 (0.53)
*asy3-1*	63	3.94 ± 1.64	0	8	118 (0.38)
*asy3-1/zyp1a-2/zyp1b-1*	98	3.82 ± 1.49	1	9	194 (0.40)
*pch2-1*	73	6.55 ± 1.93	2	10	50 (0.14)
*pch2-1/zyp1a-2/zyp1b-1*	61	5.39 ± 1.39	3	9	59.5 (0.20)
*mlh3-1*	67	4.00 ± 1.53	1	8	117 (0.35)
*mlh3-1/zyp1a-2/zyp1b-1*	54	1.94 ± 1.29	0	6	174 (0.66)
*msh5-1*	82	1.13 ± 0.978	0	4	320 (0.78)
*msh5-1/zyp1a-2/zyp1b-1*	97	0.557 ± 0.661	0	3	432 (0.89)

In addition to pairs of univalents, recombination defects such as interlocks and potential nonhomologous interactions were also observed in the *zyp1* mutants (Fig. 4 *Q*–*T*). These aberrant structures were rare, and of the 115 *zyp1* cells sampled, only seven were identified, although none were observed in the *zyp1a-1* and *zyp1-b* mutants or wild type. In one *zyp1a-2/zyp1b-1* cell, a bridge between two bivalents could clearly be identified (Fig. 4*S*). The interlocked chromosomes may be explained by the occurrence of overlapping bivalents, although bivalents are expected to be aligned on the metaphase I plate rather than perpendicular (Fig. 4*T*), and potential axial entanglements were observed in *zyp1* mutants (Fig. 2). Given the phenotype of the *ZYP1*^*RNAi*^ lines (14) and the observation of both univalent chromosomes and potential nonhomologous interactions in the *zyp1* mutants, we next sought to identify any evidence of nonhomologous chiasmata. Metaphase I chromosomes were examined by fluorescence in situ hybridization with probes labeling the 5*S* and 45*S* ribosomal DNA sequences so that all five chromosome pairs could be unambiguously distinguished (*SI Appendix*, Fig. S3). In a sample of 27 *zyp1a-2/zyp1b-1* metaphase I nuclei, six univalent pairs were observed, all of which were composed of two homologs from the same chromosome. No evidence for nonhomologous bivalent formation was observed in this sample, nor were there any clear examples of multivalent structures.

### ZYP1 Is Required for Normal Fertility.

To assess whether fertility was affected in the *zyp1* null mutants, seed counts were performed. Bonferroni-corrected pairwise *t* tests showed that the average seed set per silique was significantly higher in the wild-type plants ($\bar{x}$ = 54.0, SD = 4.70, *n* = 60) compared to the *zyp1* mutants, though for *zyp1a-1/zyp1b-2*, this effect was marginal: *zyp1a-2/zyp1b-1* ($\bar{x}$ = 50.3, SD = 4.43, *n* = 60, t = 4.45, df = 117.58, *P*adj = 9.7 × 10^−5^); *zyp1a-3/zyp1b-1* ($\bar{x}$ = 51.0, SD = 4.48, *n* = 60, t = 4.45, df = 115.98, *P*adj = 0.0023); and *zyp1a-3/zyp1b-2* ($\bar{x}$ = 51.4, SD = 5.92, *n* = 60, t = 4.45, df = 115.98, *P*adj = 0.43) (*SI Appendix*, Fig. S4). The seed set in the *zyp1a-1* and *zyp1-b* single mutants was not significantly reduced compared to the wild type: *zyp1a-1* ($\bar{x}$ = 53.3, SD = 3.83, *n* = 60, t = 0.91, df = 113.37, *P*adj = 0.326) and *zyp1b-1* ($\bar{x}$ = 52.2, SD = 4.01, *n* = 60, t = 2.26, df = 115.11, *P*adj = 0.123). Alexander staining of pollen was also performed to check the viability of pollen in *zyp1a-2/zyp1b-1*. In total, 97.3% (*n* = 744) of pollen grains were observed to be viable in the wild-type plants compared to 95.9% (*n* = 826) in the *zyp1a-2/zyp1b-1* mutant. A χ^2^ test of these samples revealed no statistically significant difference between the two genotypes (X^2^ = 1.99, df = 1, *P* = 0.16).

### ZYP1 Promotes Formation of Interference-Insensitive Chiasmata but Is Nonadditive to *asy1* and *asy3*.

To investigate the interplay between ZYP1 and the meiotic axis, triple *asy1-4/zyp1a-2/zyp1b-1* and *asy3-1/zyp1a-2/zyp1b-1* mutants were generated. The *asy1-4* single mutant is completely asynaptic, whereas short stretches of ZYP1 are observed in *asy3-1* colocalizing with foci of the Class I CO marker MLH3 (42, 55). We therefore sought to determine if the residual ZYP1 stretches in *asy3-1* affected CO formation. Metaphase I chromosome spreads were performed on single (*asy1-4* and *asy3-1*) and triple (*asy1-4/zyp1a-2/zyp1b-1* and *asy3-1/zyp1a-2/zyp1b-1*) mutant lines and rod:ring–univalent:multivalent ratios were calculated (Fig. 5). For both *asy1* compared to *asy1-4/zyp1a-2/zyp1b-1* and *asy3-1* compared to *asy3-1/zyp1a-2/zyp1b-1*, the triple mutants showed similar proportions of chromosome configurations to the single mutants, indicating that *zyp1* did not affect CO formation in *asy1-4* or *asy3-1* mutant backgrounds. Counts of total chiasmata similarly supported this observation (Table 1) and Wilcoxon ranked sum tests confirmed no significant differences between the distribution of total chiasmata in *asy1-4* and *asy1-4/zyp1a-2/zyp1b-1* (W = 1095.5, *P* = 0.906) or *asy3-1* and *asy3-1/zyp1a-2/zyp1b-1* (W = 3245.5, *P* = 0.576). Counts of total chiasmata for *asy1-4* ($\bar{x}$ = 2.65, SD = 1.32, *n* = 54) and *asy3-1* ($\bar{x}$ = 3.94, SD = 1.64, *n* = 40) appeared consistent with previously published results for *asy1-4* ($\bar{x}$ = 2.22, SD = 1.60, *n* = 63) and *asy3-1* ($\bar{x}$ = 3.33, SD = 1.49, *n* = 98) (41, 42). However, unexpectedly, a small number of nonhomologous multivalent structures were observed in both *asy1-4* (*n* = 1) (*SI Appendix*, Fig. S5*B*) and *asy3-1* (*n* = 3) (*SI Appendix*, Fig. S5 *G* and *H*) as well as *asy1-4/zyp1a-2/zyp1b-*1 (*n* = 1) (*SI Appendix*, Fig. S5*D*) and *asy3-1/zyp1a-2/zyp1b-1* (*n* = 8) (*SI Appendix*, Fig. S5*K*). Furthermore, in both *asy3-1* (*SI Appendix*, Fig. S5*H*) and *asy3-1/zyp1a-2/zyp1b-1* (*SI Appendix*, Fig. S5*L*), a small number of possible interlocks were also observed (*n* = 2 and *n* = 1, respectively). Most surprisingly, in both *asy3-1* (*SI Appendix*, Fig. S5*F*) and *asy3-1/zyp1a-2/zyp1b-1* (*SI Appendix*, Fig. S5*L*), small chromosome fragments were observed (*n* = 7 and *n* = 6, respectively). Quantification of aberrant chromosome structures scored at metaphase I from all genotypes is presented in *SI Appendix*, Table S2.

In the *Arabidopsis pachytene checkpoint 2* (*pch2*) mutant, MLH3 foci colocalized with ZYP1, often clustered in short stretches (43). We therefore investigated whether elimination of ZYP1 affected CO formation in *pch2-1*. Total chiasmata per cell were significantly reduced in *pch2-1/zyp1a-2/zyp1b-1* ($\bar{x}$= 5.39, SD = 1.39, *n* = 61) compared to *pch2-1* ($\bar{x}$= 6.55, SD = 1.93, *n* = 73) (Wilcoxon rank sum test: W = 3024.5, *P* = 3.00 × 10^−4^) (Table 1). In addition, univalents and rod bivalents were more frequent in *pch2-1/zyp1a-2/zyp1b-1* compared to the *pch2-1* single mutant (Fig. 5). A small number of multivalent chromosomes were identified in *pch2-1/zyp1a-2/zyp1b-1* (*n* = 4) (*SI Appendix*, Fig. S6*D*), but none were identified in *pch2-1*, although in a small number of cells for both *pch2-1* (*n* = 2) and *pch2-1/zyp1a-2/zyp1b-1* (*n* = 2), some connections were observed between bivalents (*SI Appendix*, Figs. S6 *B*, *C*, and *F*). Therefore, these data indicate that ZYP1 promotes formation of a proportion of COs in the *pch2-1* mutant.

To examine the role of ZYP1 in the Class I CO interference-sensitive pathway, *zyp1a-2/zyp1b-1* was crossed with *msh5-1* (45) and *mlh3-1* (44), and metaphase I chromosome configurations were then scored (Fig. 5 and *SI Appendix*, Fig. S7). This revealed that univalents and rod bivalents were more common in *msh5-1/zyp1a-2/zyp1b-1* and *mlh3-1/zyp1a-2/zyp1b-1* than in the *msh5-1* or *mlh3-1* single mutants (Fig. 5). Similarly, the mean chiasmata per cell appeared reduced in both triple mutants compared to single mutants: *msh5-1* (1.13 ± 0.98, *n* = 82) versus *msh5-1/zyp1a-2/zyp1b-1* (0.56 ± 0.66, *n* = 97), and *mlh3-1* (4.00 ± 1.53, *n* = 67) versus *mlh3-1/zyp1a-2/zyp1b-1* (1.94 ± 1.29, *n* = 53) (Table 1). Wilcoxon rank sum tests confirmed the observed reductions in chiasmata were significant for both *msh5* compared to *msh5-1/zyp1a-2/zyp1b-1* (W = 5350, *P* = 1.85 × 10^−5^) and *mlh3-1* compared to *mlh3-1/zyp1a-2/zyp1b-1* (W = 3012, *P* = 3.29 × 10^−11^). For both *msh5-1/zyp1a-2/zyp1b-1* and *mlh3-1/zyp1a-2/zyp1b-1*, the proportional reduction in chiasmata compared to the single mutants was similar, being 52% in *msh5-1/zyp1a-2/zyp1b-1* compared to *msh5-1* and 51% in *mlh3-1/zyp1a-2/zyp1b-1* compared to *mlh3-1*. Interestingly, the *mlh3-1/zyp1a-2/zyp1b-1* to *mlh3-1* comparison showed the largest decrease in chiasma frequency observed for all *zyp1* recombination pathway triple mutants at ∼2 per cell, which was markedly more than that observed in *zyp1a-2/zyp1b-1* compared to the wild type (∼0.8). As the loss of ∼2 chiasmata per cell is greater than the expected maximum contribution from the Class II CO pathway (1.13) in *mlh3-1*, these results suggest that ZYP1 is promoting both Class I and Class II COs in these mutant backgrounds.

### ZYP1 Limits Interference-Sensitive COs and Promotes CO Interference.

**Fig. 6.**
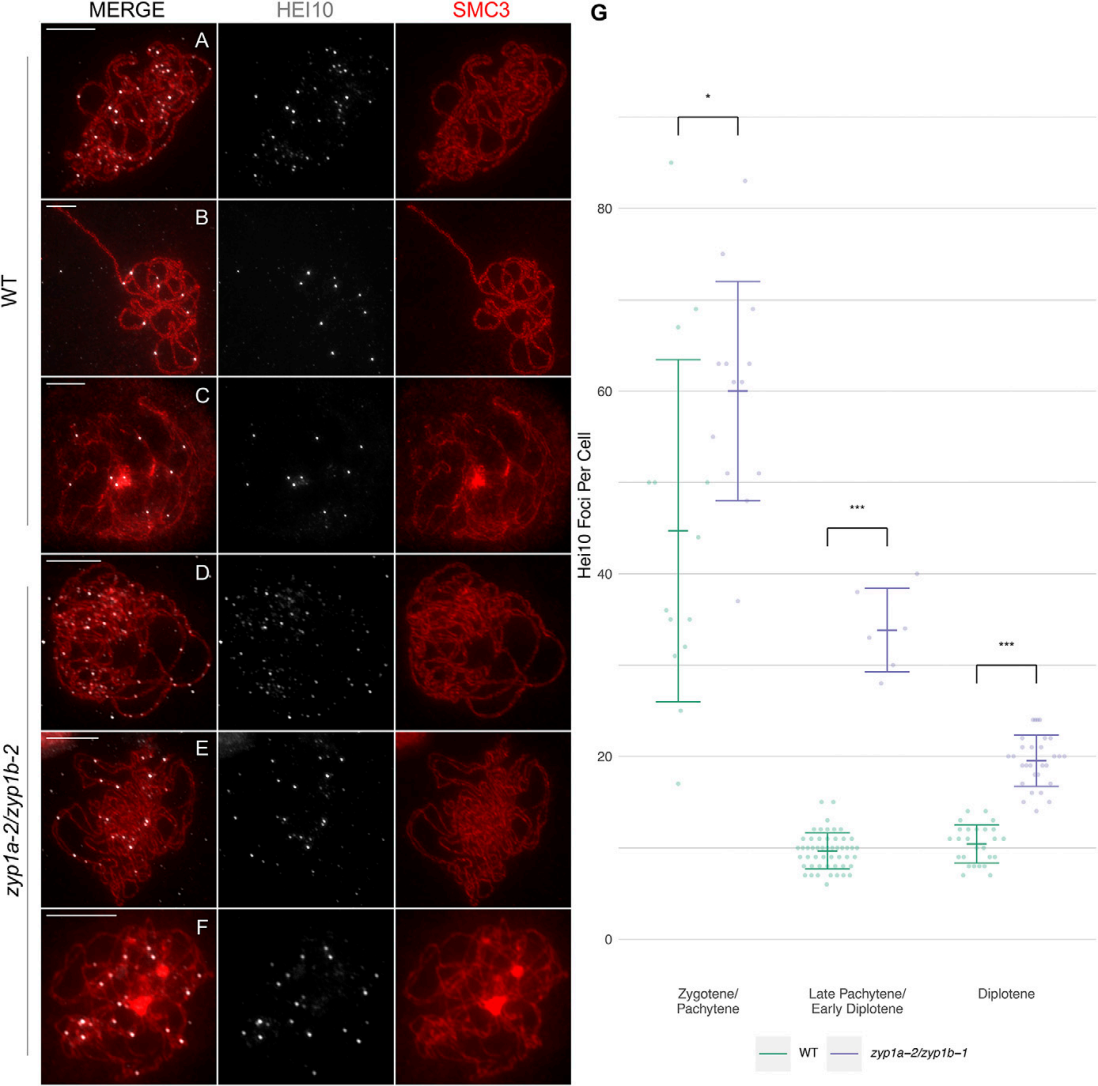
Higher numbers of HEI10 foci persist in the absence of ZYP1. Coimmunofluorescence of HEI10 and SMC3 on meiotic prophase I chromosome spreads. (*A* and *D*) Zygotene/early pachytene (and analog). (*B* and *E*) Late pachytene and “pachytene”’/diplotene transition. (*C* and *F*) Diplotene. (Scale bars, 10 µm.) (*G*) Counts of HEI10 foci per cell for the wild type and *zyp1a-2/zyp1b-1*. *P < 0.05 and ***P < 0.001 for a two-sample Wilcoxon ranked sum test.

Though there was little difference in the number of chiasmata between the *zyp1* mutants and wild type, it was possible that these counts would underestimate the number of COs (as adjacent noninterfering COs might be scored as a single chiasma). To investigate the effect of *zyp1* on the Class I interference-sensitive CO pathway, HEI10 foci numbers were compared between the mutant and wild type (56). HEI10 is the *A. thaliana* ortholog of *S. cerevisiae Zip3* and promotes formation of Class I COs (57). In the wild type, as previously reported, a large number of foci were observed at late zygotene/early pachytene ($\bar{x}$ = 44.7, SD = 18.7, *n* = 14) (Fig. 6 *A* and *G*).

This stage could be identified by a combination of axis morphology and HEI10 localization which showed numerous foci of variable sizes and intensities. By contrast, ∼10 HEI10 foci were typically much larger and uniform in intensity at late pachytene ($\bar{x}$ = 9.76, SD = 1.98, *n* = 51) (Fig. 6 *B* and *G*) and diplotene ($\bar{x}$ = 10.4, SD = 2.08, *n* = 26) (Fig. 6 *C* and *G*). Similar to wild type, in *zyp1a-2/zyp1b-1*, a large number of HEI10 foci of variable size and intensity were observed at “zygotene/early pachytene” ($\bar{x}$ = 60.0, SD = 12.0, *n* = 13) when the cells showed extensive aligned axes (Fig. 6 *D* and *G*). Wilcoxon rank sum test showed a statistically significant difference between the number of foci between the wild type and the *zyp1a-2/zyp1b-1* mutants at this stage (W = 40.5, *P* = 0.015). However, identification of cells analogous to late pachytene was much less clear. In particular, cells with bright, uniform HEI10 foci such as those observed in wild type were not apparent. Comparable nuclei which appeared to be at the transition into diplotene were identified, but these cells were infrequent, possibly suggesting the transient nature of this stage (Fig. 6 *E* and *G*). In general, the chromosome axes in these “late pachytene”/diplotene transition nuclei were aligned much less closely than the earlier stages, but the HEI10 foci appeared more regular in size and intensity compared to the earlier cells. An average of ∼30 HEI10 foci were identified in these nuclei ($\bar{x}$ = 33.8, SD = 4.58, *n* = 6). A Wilcoxon rank sum test confirmed that the difference observed between the HEI10 foci counted in wild type for late-pachytene cells was significantly different from that counted in the *zyp1a-2/zyp1b-1* mutant pachytene/diplotene transition stage (W = 0, *P* = 6.15 × 10^−5^). In the *zyp1a-2/zyp1b-1* mutants, unlike the wild type, the number of HEI10 foci appeared to be further reduced during diplotene and by the time nuclei appeared characteristically diplotene-like, there were approximately ∼20 HEI10 foci ($\bar{x}$ = 19.5, SD = 4.58, *n* = 29) (Fig. 6 *F* and *G*). A Wilcoxon rank sum test confirmed this was significantly different than that observed in wild type diplotene nuclei (W = 1, *P* = 2.24 × 10^−10^).

**Fig. 7.**
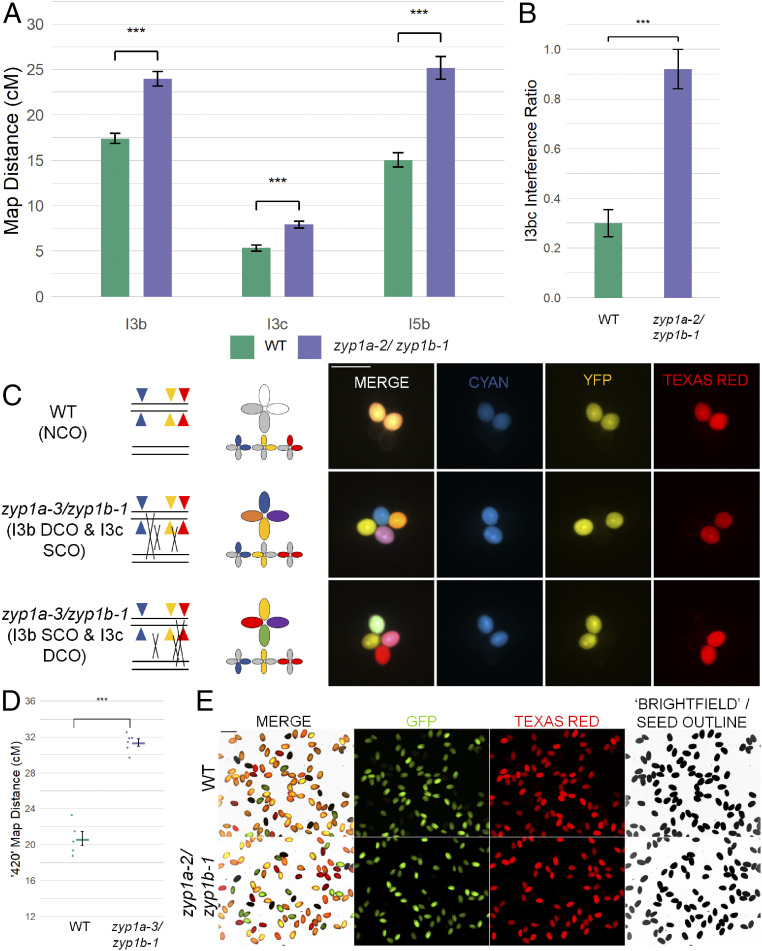
Recombination and double COs increase in *zyp1*. (*A*) Genetic map distance (centi-Morgan) determined by recombination in pollen FTLs in three intervals. (*B*) Interference ratio calculated in the I3bc double interval. (*C*) Illustration and examples of double COs observed in *zyp1* fluorescent pollen tetrads and an NCO in wild type. (*D*) Genetic map distance (cM) determined by fluorescent seed-based assay of the 420 interval. (*E*) An example of a field of view from the fluorescent seed recombination assay.

As chiasmata were slightly reduced, but the numbers of HEI10 foci were approximately twofold higher at diplotene in the *zyp1* mutants compared to wild type, a genetic approach using fluorescence-tagged lines (FTL) was employed to test the rate of recombination and CO interference. Fluorescent pollen tetrads were scored for the I3bc and I5b intervals (48). In all intervals examined, the map distance was significantly increased by 1.3- to 1.7-fold compared to wild type: I3b (WT: 17.43 centi-Morgan [cM], SE = 0.57 cM) (*zyp1a-3/zyp1b-1*: 23.98 cM, SE = 0.81 cM, *P* = 1.97 × 10^−11^); I3c (WT: 5.34 cM, SE = 0.34 cM) (*zyp1a-3/zyp1b-1*: 7.93 cM, SE = 0.38 cM, *P* = 1.73 × 10^−7^); and I5b (WT: 15.06 cM, SE = 0.78 cM) (*zyp1a-3/zyp1b-1*: 25.18 cM, SE = 1.26 cM, *P* = 4.22 × 10^−12^) (Fig. 7 and *SI Appendix*, Tables S2–S5).

As well as a marked increase in the map distance for all three intervals, the I3bc double interval revealed that CO interference had been virtually abolished in *zyp1a-3/zyp1b-1* (intra-interference ratio: WT = 0.3, SE = 0.055; *zyp1a-3/zyp1b-1* = 0.92, SE = 0.079, *P* = 4.62 × 10^−11^; in which a value of 0 = strong interference and 1 = no interference). This was supported by observations of tetrads showing up to three adjacent COs within the I3bc intervals (I3b double cross-over [DCO] with I3c single cross-overs [SCOs] and vice versa) which was never observed in the wild type (Fig. 7 *B* and *C*). Further supporting the observation that CO interference was substantially reduced in *zyp1a-3/zyp1b-1*, estimations of interference from single-interval tetrad data also were calculated. For I3b and I5b, the ratio of Observed DCOs/Expected DCOs (under the assumption of no interference) were increased in *zyp1* compared to the wild type (I3b: WT = 0.17, *zyp1a-3/zyp1b-1* = 0.74; I5b: WT = 0.23, *zyp1a-3/zyp1b-1* = 0.57). For both the wild type and *zyp1a-3/zyp1b-1*, however, a χ^2^ test showed that the counts of tetrads differed significantly from that expected without CO interference (I3b: WT *P* = 3.69 × 10^−8^, *zyp1a-3/zyp1b-1 P* = 0.018; I5b: WT *P* = 0.0024, *zyp1a-3/zyp1b-1 P* = 0.0071) (*SI Appendix*, Table S6). I3c interinterval interference ratios did not differ between *zyp1a-3/zyp1b-1* and the wild type (I3c: WT = 0.30, *zyp1a-3/zyp1b-1* = 0.34), although very few DCOs (<10) were observed and expected in both the mutant and wild type. Taken together, these results indicate that ZYP1 mediates CO interference in *Arabidopsis*.

As the pollen FTLs only measure the recombination products from male meiosis, we also employed the 420 seed-based FTL interval to jointly assay recombination from male and female meiosis (47). Map distances were increased in *zyp1a-3/zyp1b-1* ($\bar{x}$= 31.40 cM, SE = 0.41 cM, *n* = 6) compared to the wild type ($\bar{x}$= 20.66 cM, SE = 0.81 cM, *n* = 5) (Fig. 7 *D* and *E* and *SI Appendix*, Table S7). A *t* test confirmed this difference was statistically significant (t = 11.882, df = 6.0257, *P* = 2.085 × 10^−5^). These results showed that recombination had increased by 1.52-fold, consistent with the observed increases in the pollen intervals, suggesting a consistent effect upon male and female meiosis.

## Discussion

The SC transverse filament proteins ZYP1a and ZYP1b are encoded by two tandemly duplicated genes that maintain CO assurance and the fidelity of recombination in *Arabidopsis* (14). Stable *zyp1a* and *zyp1b* double knockouts using CRISPR/Cas have now been generated that reveal previously unknown roles for the SC in *Arabidopsis*.

In the *zyp1* null mutants, the rare occurrence of univalent chromosomes indicated a CO control defect leading to loss of CO assurance, consistent with previous observations in *ZYP1*^*RNAi*^ knockdown lines (14). In contrast to the *ZYP1*^*RNAi*^ lines, we did not find compelling examples of multivalents, although a small number of cells exhibited ectopic recombination. The reason for this is unclear; however, we cannot exclude the possibility that a low level of ZYP1 protein remained in the RNAi lines which was not detected cytologically. Alternatively, the nonhomologous associations may have occurred due to small undetected chromosome translocations arising during the transformation process or a combination of environmental factors, such as elevated temperature during flowering and depletion of ZYP1 (58). Cytological analysis in the *ZYP1*^*RNAi*^ lines failed to detect any significant differences in chiasma distribution, leading to the suggestion that CO interference had not been affected. However, more recent studies using Class I CO markers such as HEI10/MLH3 have shown that in some mutants that disrupt CO patterning, closely spaced COs can occur that would be “invisible” to chiasma analysis (43). To avoid this potential issue, we utilized a genetic assay to quantify CO interference based on FTLs in the pollen *quartet* mutant (48). This analysis revealed a large decrease in CO interference in *zyp1 (*ratio *=* 0.92) compared to wild type (ratio = 0.3). An increase in recombination in the mutant was also found using seed-based markers that, unlike pollen markers which only assess male meiotic recombination, report both male and female meiosis (47). Immunolocalization of the Class I CO marker HEI10 in wild type *Arabidopsis* shows that the protein initially forms over 100 foci associated with chromosome axes at leptotene (56). As prophase I progresses, the foci associate with the central region of the SC where their numbers decrease such that by late pachytene/diplotene, ∼10 foci remain, corresponding to the number of Class I COs (56). In wild type, we observed ∼45 HEI10 foci at late zygotene that decreased to ∼10 at late pachytene and persisted during diplotene. This higher number of HEI10 foci at zygotene compared to diplotene likely reflects HEI10 localization at synapsis initiation sites which are also reported to exhibit patterning (56), but only a proportion of which correspond to future COs. This is supported by a study in the closely related species *Brassica oleracea* which showed that at zygotene, 86% of HEI10 foci colocalized with synapsis initiation sites (43). In *zyp1*, we also observed a progressive reduction in HEI10 foci between late zygotene through diplotene although the final number was twofold greater in *zyp1* (∼20 foci) compared to wild type (∼10). This observation supports the genetic analysis of *zyp1* that indicated an increase in recombination frequency. Elevated levels of COs, including double COs, appear to persist in *zyp1* indicative of a defect in CO control. Studies in *C. elegans* suggest that the SC TF proteins limit COs and attenuate CO interference (40). However, studies in *S. macrospora* indicate that interference acts on a large number of recombination initiation events (∼80) giving rise to a smaller number (∼45 to 50) of patterned, evenly spaced, synapsis initiation sites and CO designations, thereby arguing that CO patterning is established prior to SC formation (35, 57). Studies in *S. cerevisiae* also support a model of early CO designation (6, 32, 59). In addition, further evidence from *S. cerevisiae* suggests that Topoisomerase II plays an important role in mediating CO interference by relieving mechanical stress at a CO-designated site and a signal is transmitted along the chromosome axes, so that recombination intermediates adjacent to a CO are repaired as non-COs (35). Analyses in *Arabidopsis* also supports a model whereby a smaller number of synapsis initiation sites plus CO-fated intermediates emerge from a large number of initiating DSBs (49). Our data could be consistent with ZYP1 transmitting an interference signal in a distance-dependent manner by removing closely spaced non-CO–fated recombination intermediates. However, as the meiotic programs in plants, fungi, and mammals appear very similar (54), it seems perhaps more likely that ZYP1 is required to ensure the correct fate of an interference-dependent patterned array of recombination complexes. The resultant loss of SC in the *zyp1* mutant leads to a defect in fate implementation, resulting in an increase in COs which are less closely spaced than in the wild type.

Class II noninterfering COs account for ∼15% of COs in wild type, and the additional COs in the *Arabidopsis* anti-recombination mutants (*fancm*, *mhf1*/*mhf2*, *recq4a*/*recq4b*, *figl1*, and *flip*) (60–65) are processed down this pathway. To determine whether the increase in recombination and loss of interference in *zyp1* was due to an increase in Class II COs, the *zyp1* mutant was crossed with Class I CO mutants *msh5* (45) and *mlh3* (44). The *zyp1* mutant did not restore chiasmata to wild type levels, either in *msh5* or *mlh3,* which are restored in crosses with the anti-recombination mutants (60–65). In *msh5/1/zyp1* triple mutants, chiasma counts revealed ∼50% of the Class II COs were abolished (0.56 chiasmata per cell were observed, compared to 1.13 in *msh5*), suggesting that ZYP1 was promoting the formation of Class II COs rather than preventing them. This suggests that the integrity of the SC is required to ensure the formation of both classes of CO in *Arabidopsis*.

To gain an insight into the role of ZYP1 as a structural component of the SC, a cytological analysis using super-resolution microscopy was performed. In the absence of ZYP1, homologs coaligned at a distance of ∼328 nm, compared to ∼231 nm in wild type, consistent with refs. 25 and 28. In *zyp1*, the distance between the axes was variable, and entanglements were also observed, which may have led to a small number of ectopic chromosome associations observed at metaphase I. Previous studies have shown that early events in interhomolog recombination mediate coalignment of the chromosome axes, as reviewed in ref. 54. This involves the establishment of a series of cytologically visible bridges that link the axes at a distance of ∼400 nm (66). These bridges occur at sites of recombination and are marked by recombination proteins (5). A detailed study in *S. macrospora* shows that as prophase I progresses, the axes become progressively more closely aligned through shortening of the interaxis bridges. Once the axes become juxtaposed at a distance of ∼100 nm, installation of the SC central region occurs. Analysis reveals that the interaxis bridges in *S. macrospora* contain axis proteins and recombination complexes that undergo a series of transitions to ultimately reposition complexes containing the ZMM proteins from the axis to the central region between the closely apposed axes. Interestingly, the TF protein Sme4 is required for this repositioning prior to installation of the SC (5). Our data in *Arabidopsis* indicates that ZYP1 loading is initiated at evenly spaced (∼290 nm) ASY1-labeled interaxis bridges. Synapsis proceeds then by “filling in the gaps” between the axial bridges, which is concomitant with the depletion of ASY1 by PCH2/p31Comet from the synapsed axial region (43, 67). This model is consistent with analysis of the barley SC structure by super-resolution microscopy and may reflect conservation in plants (28). Previous studies have shown that in an *asy1* mutant, ZYP1 does not load onto the meiotic chromosomes, but in an *asy3* mutant in which ASY1 forms discreet foci along the axis rather than a continuous signal, synapsis is nucleated, although ZYP1 polymerization is limited to short stretches (42, 55). Moreover, in *asy1*, a small number of COs form at the distal ends of the chromosomes, but these do not initiate synapsis, suggesting that interhomolog recombination intermediates alone are not sufficient to promote ZYP1 loading (55, 68). Taken together, these observations suggest that ASY1 labeled axial bridges are a prerequisite to enable ZYP1 loading.

It appears that in *zyp1*, early steps in axis coalignment occur, possibly as normal, suggesting the establishment of interaxis bridges. However, the normal transition to close coalignment is disrupted, possibly because, like Sme4, ZYP1 has a role in this process (5). The association of ASY1 with the homolog axes and possibly the axial bridges persists, as its depletion by PCH2/p31Comet requires ZYP1 localization (43, 67). A result of these events could be that the normal spatial redistribution of the recombination complexes is aberrant, and as a consequence, normal patterned CO formation is perturbed. Our observations in *zyp1* are similar to those in rice mutants lacking the TF protein ZEP1 in which the axes remain largely coaligned at mid/late prophase I. PAIR2/ASY1 persists on the axes and COs are elevated (16).

In conclusion, ZYP1 plays a key role in CO control in *Arabidopsis*. Loss of the protein results in failure to form an obligatory CO between each homolog pair. Crucially, the protein is essential to ensure normal interference-dependent CO patterning.

## Supplementary Material

Supplementary File

## Data Availability

All study data are included in the article and/or *SI Appendix*.
