# Supplementary material for: ZYP1 is required for obligate cross-over formation and cross-over interference in Arabidopsis
Source: Proc Natl Acad Sci U S A. 2021 Mar 29;118(14):e2021671118. doi: 10.1073/pnas.2021671118 (PMC8040812; doi:10.1073/pnas.2021671118)
Supplement: Supplementary File [file pnas.2021671118.sapp.pdf]

SI Appendix

Supplemental methods

Validation of zyp1 mutants

All primers used for genotyping are presented in Table S1. The sgRNA expression cassette was and transformed into the respective *zyp1a-1* and *zyp1b-1* mutants by floral-dipping (1) and transformants were selected on 50 µM phosphinothricin. Mutations were confirmed by sequencing amplicons (Eurofins) and homozygous plants carrying frameshift mutations were used for analysis. T-DNA insertions were confirmed by PCR as previously described (2). *zyp1a-2* and *zyp1a-3* were genotyped by CAPS as the mutation abolished a *Hin*dIII site and *zyp1b-2* was genotyped by amplicon sequencing.

Super-resolution microscopy and analysis

Image capture and reconstruction for SIM was performed using a Zeiss ELYRA PS1 microscope (John Innes Centre) and OMX microscope (Applied Precision) at the University of Dundee with Imaris software (Oxford Instruments). Images were imported into NIS-elements software (Nikon) for measuring distances between ASY1-labelled axial bridges. Three independent cells and 18 counts were recorded. The distance between lateral elements/axes was measured for a maximum intensity projection of the SMC3 channel using ImageJ and R. For each cell (wild type, n = 2; *zyp1a-2/zyp1b-1,* n = 3), random rectangular regions 50 pixels wide were manually annotated with perpendicular lines bisecting the two juxtaposed axes and repeated 20 times. The plot profile of fluorescence intensity was then exported and analysed in R. For each line an interpolating spline anchored was fitted (*spline* function - *stats* package). Fitted splines were used to predict a denser array of values (*predict* – *stats* package). The turning points corresponding to the centre of each lateral element were identified (*findpeaks* – *pracma* package), and the distance between the centre of the axes was calculated.

Chiasmata counts

Metaphase I chromosome spread preparations were prepared from ethanol: acetic acid (3:1) fixed material. Chiasma counts were based on the shape of bivalents and fluorescence *in situ* hybridization was performed with the 5*S* and 45*S* rDNA probes (3).

Pollen FTL and viability analysis

*zyp1a-3/zyp1b-1/*I3bc *qrt1-2*/I5ab *qrt1-2* crosses were sown, following which F1 and F2 generations were allowed to self. F3 plants were then genotyped to identify *zyp1a-3/zyp1b-1* mutants and segregated wild-type controls. These were screened to identify *qrt1-2* plants with the correct hemizygous fluorescence patterns. For the I3bc cross, 3 mutants and 1 wild type plant were recovered with the correct pattern (CYR/+++). For I5ab no plants were recovered to analyse all three intervals (RYC/+++) but 1 wild type plant and 1 mutant plant with appropriate I5b fluorescence patterns was recovered (+YC/+++). Fluorescent tetrad analysis was carried out as previously described (4). Pollen quartets were imaged on a Nikon Ni-E fluorescence microscope using a 10x objective lens and CFP (Ex: 436/20 nm, Em: 480/40 nm), YFP (Ex: 500/20 nm, Em 535/30 nm) and mCherry (Ex: 560/40 nm, Em: 630/75 nm) filters. Pollen quartets were classified into the 12 possible groups by manual scoring. Statistical analysis was carried out using Stahl Lab Online tools (https://elizabethhousworth.com/StahlLabOnlineTools/). For estimations of interference from single interval tetrad data the "Better way" method was used. Z tests were used to estimate significance from population summary statistics between mutant and wild-type map distances and interference ratios (Berchowitz and Copenhaver 2008). The ratio of viable to non-viable pollen grains for the *zyp1a/zyp1b* double mutants was determined using Alexander staining (5).

Seed FTL analysis

zyp1a-2/zyp1b-1 was crossed with the fluorescent seed marker line 420 (6). The F1 generation was selfed and seeds were screened to select plants carrying both RFP and GFP transgenes. F2 plants were then genotyped for *zyp1a-2/zyp1b-1* mutants and segregated wild-type controls that were screened to identify plants with the correct hemizygous fluorescence patterns (RG/++). Seeds were collected from these plants (wild type, n = 5 and *zyp1a-2/zyp1b-1,* n = 6) for scoring. The Nikon Ni-E fluorescence microscope was used with a 1x objective lens and the following filters; 90/10 (Reflection/Transmission) Beamsplitter ('Brightfield' / Seed outline), GFP (Ex: 470/40 nm, 525/50 nm) and mCherry (Ex: 560/40 nm, Em: 630/75 nm). Seed scoring was automated in a method adapted from (7). An ImageJ macro was used to detect and measure the mean RFP and GFP fluorescence intensity of every seed in each field of view for all images per plant. A rolling ball background subtraction was applied to all channels (*subtract background*: radius of 50 pixels for 'brightfield' channel seeds appeared as dark objects against a light background). The 'brightfield' image was then converted to binary (*Make Binary)*, the binary mask was eroded to reduce contacts between adjacent seeds (*Erode)* and the image was then watershed to split groups (*Watershed)*. Following this, seed outlines were detected (filtering for size and circularity) and added to the ROI manager (*Analyse Particles:* size 1200-4500, circularity 0.4-01). The fluorescence of ROIs was then measured in red and green channels. For each individual, fluorescence measurements were compiled in R, normalised against the maximum fluorescence intensity observed. Histograms of red and green fluorescence were then plotted and a threshold of fluorescent intensity for each channel was manually determined and inputted. Finally, total seeds and recombinant single colour only fluorescent seeds (Red only Fluorescent / Green Only Fluorescent) were counted, and map distance determined as previously described (6, 7). This process enabled rapid and accurate measurements of >1000 seeds per individual.

Statistical analysis

All statistical analyses (excluding pollen quartet analysis) were performed using R. T tests (*t.test)* and Wilcoxon Rank-Sum Tests (*wilcox.test),* Chi Squared tests (*Chisq.test*)*,* Fligner-Killeen tests (*fligner.test*), multiple testing corrections (*p.adjust*) and z test p value calculations (*pnorm*) were performed using the given functions from the *stats* package. G tests (*G.Test*) were performed using the *DescTools* package. Multiple testing corrections were applied for Wilcoxon Rank-Sum Tests of chiasmata counts between the wild type and the three *zyp1* double mutants (n = 3) and for t tests of seed counts between wild type and all five *zyp1* mutants (both single mutants and double mutants) (n = 5).

```
ZYP1a cds      251 AGAACGCTTTTAATGAGAATGCTAAGCTCAGAGTTAGGAAAAAGGAAGAT   300
                   |||||||||||||||||||||||||||||||||||||||||||||||
zyp1a-2         39 AGAACGCTTTTAATGAGAATGCTAAGCTCAGAGTTAGGAAAAAGGAA---    85

ZYP1a cds      301 GAGAAGCTTTGGAGAGGATTGGAGTCTAAGTTCTCTTCAACAAAGACTCT   350
                              |||||||||||||||||||||||||||||||||||||||
zyp1a-2         86 -----------GAGAGGATTGGAGTCTAAGTTCTCTTCAACAAAGACTCT   124

ZYP1a cds      351 GTGTGATCAACTTACAGAGACTCTGCAGCATTTGGCTTCCCAAGTTCAGG   400
                   ||||||||||||||||||||||||||||||||||||||||||||||||||
zyp1a-2        125 GTGTGATCAACTTACAGAGACTCTGCAGCATTTGGCTTCCCAAGTTCAGG   174


ZYP1a cds      213 GTCAATGGAACATGTCTATGCCTTAGAGGAGAAACTGCAGAACGCTTTTA   262
                   ||||||||||||||||||||||||||||||||||||||||||||||||||
zyp1a-3        165 GTCAATGGAACATGTCTATGCCTTAGAGGAGAAACTGCAGAACGCTTTTA   214

ZYP1a cds      263 ATGAGAATGCTAAGCTCAGAGTTAGGAAAAAGGAAGATGAGAAGCTTTGG   312
                   ||||||||||||||||||||||||||||||||||||||           |
zyp1a-3        215 ATGAGAATGCTAAGCTCAGAGTTAGGAAAAAGGAAGAT-----------G   253

ZYP1a cds      313 AGAGGATTGGAGTCTAAGTTCTCTTCAACAAAGACTCTGTGTGATCAACT   362
                   ||||||||||||||||||||||||||||||||||||||||||||||||||
zyp1a-3        254 AGAGGATTGGAGTCTAAGTTCTCTTCAACAAAGACTCTGTGTGATCAACT   303


ZYP1b cds        1 ATGCAGAAGTTAGGGTTTCCGGCGATGAAGAGCTTTGATCAACTCCGATC    50
                                  |||||||||||||||||||||||||||||||||||
zyp1b-2          1 ---------------TTtCcGgCGaTGAaGAGCTTtGatcAACTCCgaTC    35

ZYP1b cds       51 GTTGCCTGGA-TCGGCGAAGACGTACTTCTTCTCCACTCGTCCGCCTCAG    99
                   |||||||||| |||||||||||||||||||||||||||||||||||||||
zyp1b-2         36 GTTGCCTggaTTCGGCGAAGACGTACTTCTTCTCCACTCGTCCGCCTCAG    85

ZYP1b cds      100 GATTCCGTCTCCTCCGGAAGTTTCTCGAATTTGAAACTCACAGCAGAGAA   149
                   ||||||||||||||||||||||||||||||||||||||||||||||
zyp1b-2         86 GATTCCGTCTCCTCCGGAAGTTTCTCGAATTTGAAACTCACAGCAG----   131
```

Fig. S1. Pairwise alignment between reference coding sequence (cds) and sequenced amplicons of *zyp1* CRISPR/Cas mutations. Reference sequence numbers show position relative to start codon. CRISPR/Cas9 derived indels highlighted in yellow and *Hin*dIII restriction sites highlighted in blue.

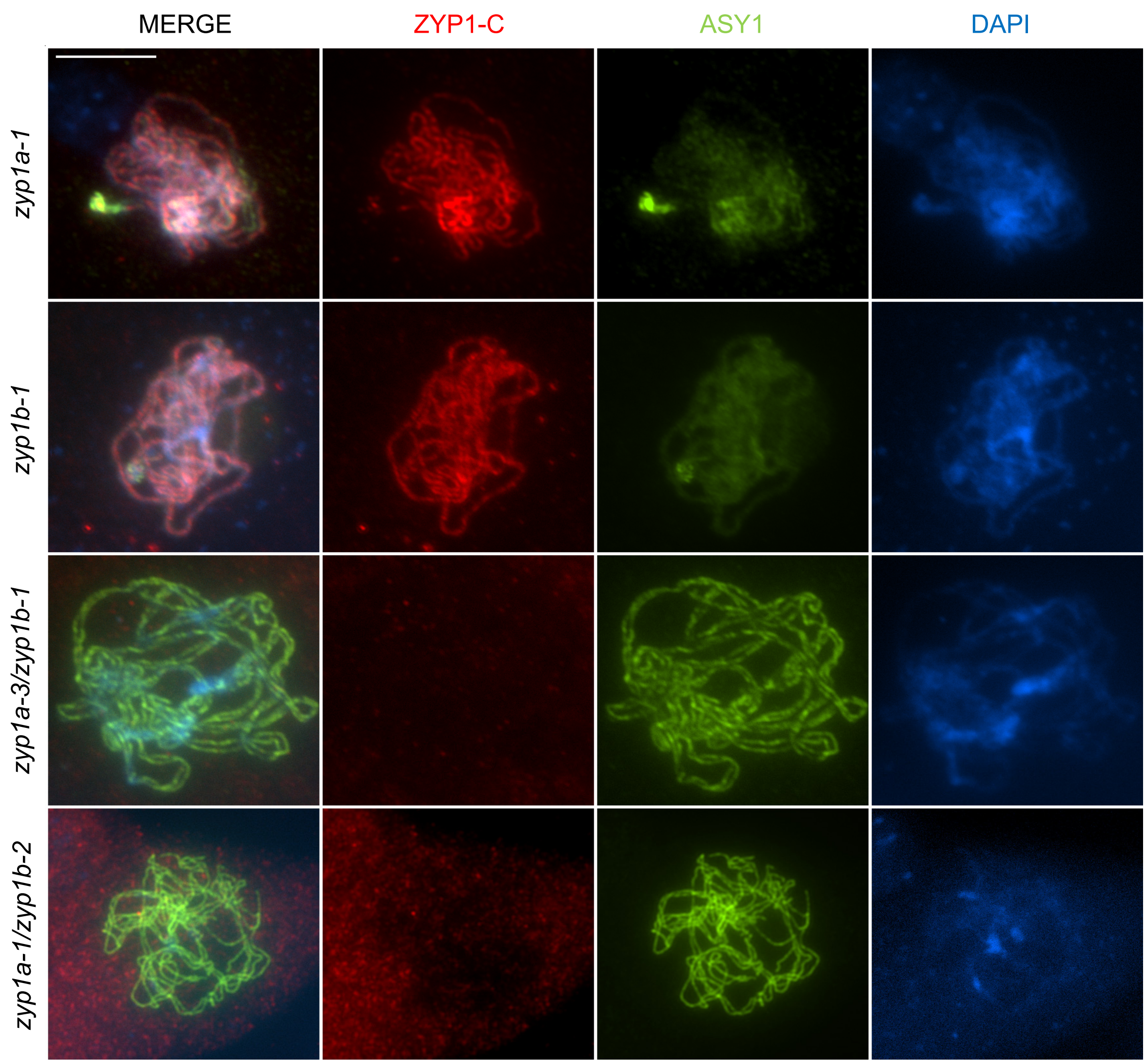

Fig. S2. *zyp1a-3/zyp1b-1* and *zyp1a-1/zyp1b-2* corroborate the *zyp1a-2/zyp1b-1* phenotype. Co-immunofluorescence of ASY1 (full length) and ZYP1 (C-terminal region) on meiotic prophase stage I chromosome spread preparations. Scale bar = 10μm.

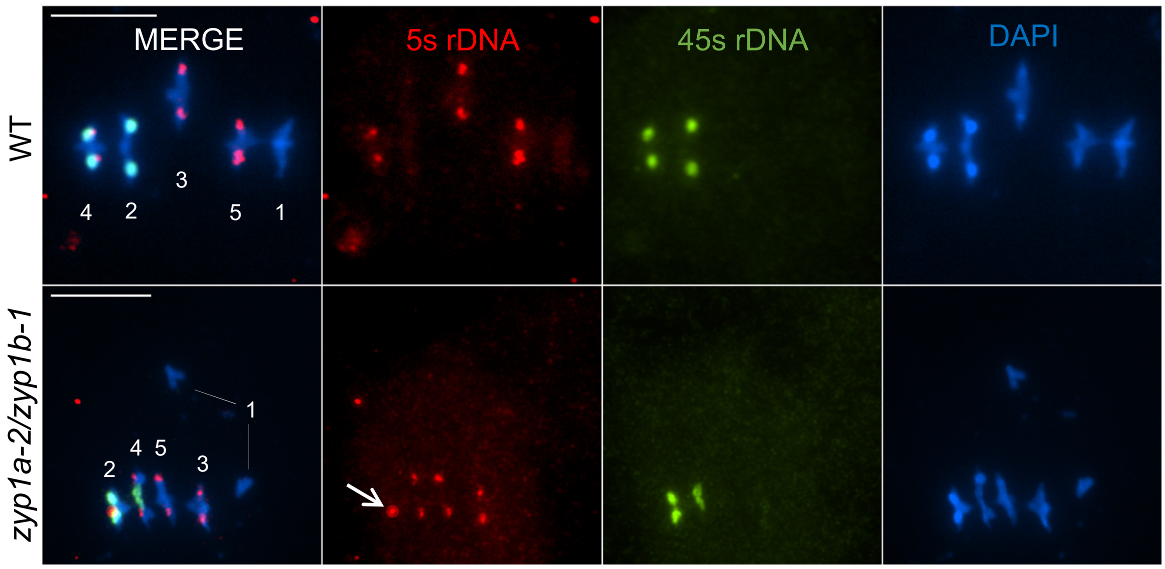

Fig. S3. Fluorescence *in situ* hybridization of 5*S* and 45*S* ribosomal DNA probes on meiotic metaphase I spreads showed no evidence of non-homologous CO formation *zyp1a-2/zyp1b-1* mutants. Chromosome identities are indicated by numbers based on 5*S* (red) and 45*S* (green) signals on DAPI (blue) stained DNA. Four homologous bivalents and a pair of homologous univalents is shown for *zyp1a-2/zyp1b-1*. White arrow indicates background staining overlapping with the *zyp1a-2/zyp1b-1* cell preparation. Scale bars = 10μm.

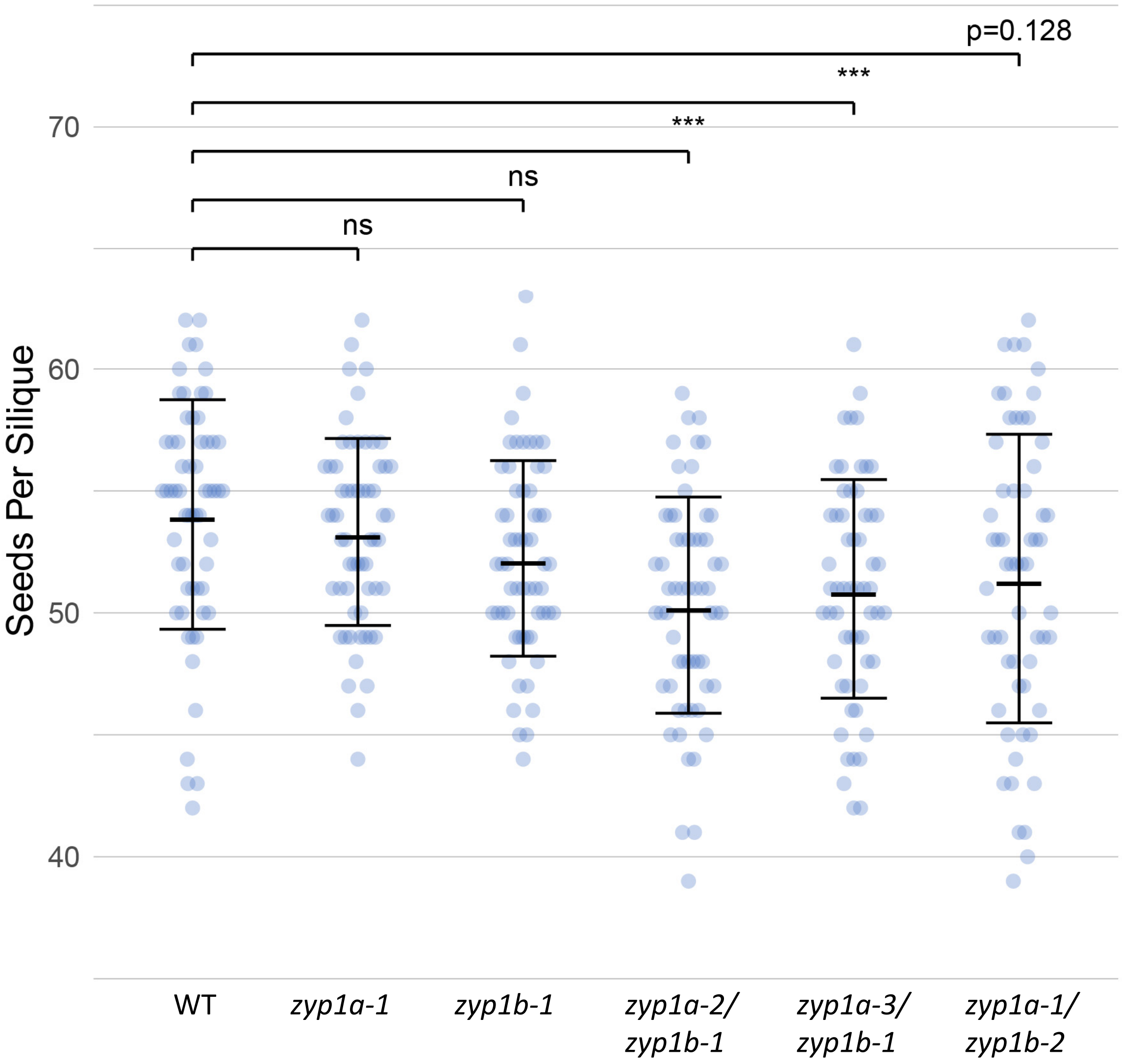

Fig. S4. Fertility test of *zyp1* mutants by seed counts. The numbers of seeds per silique in wild type compared to *zyp1* single mutants and *zyp1* double mutants.

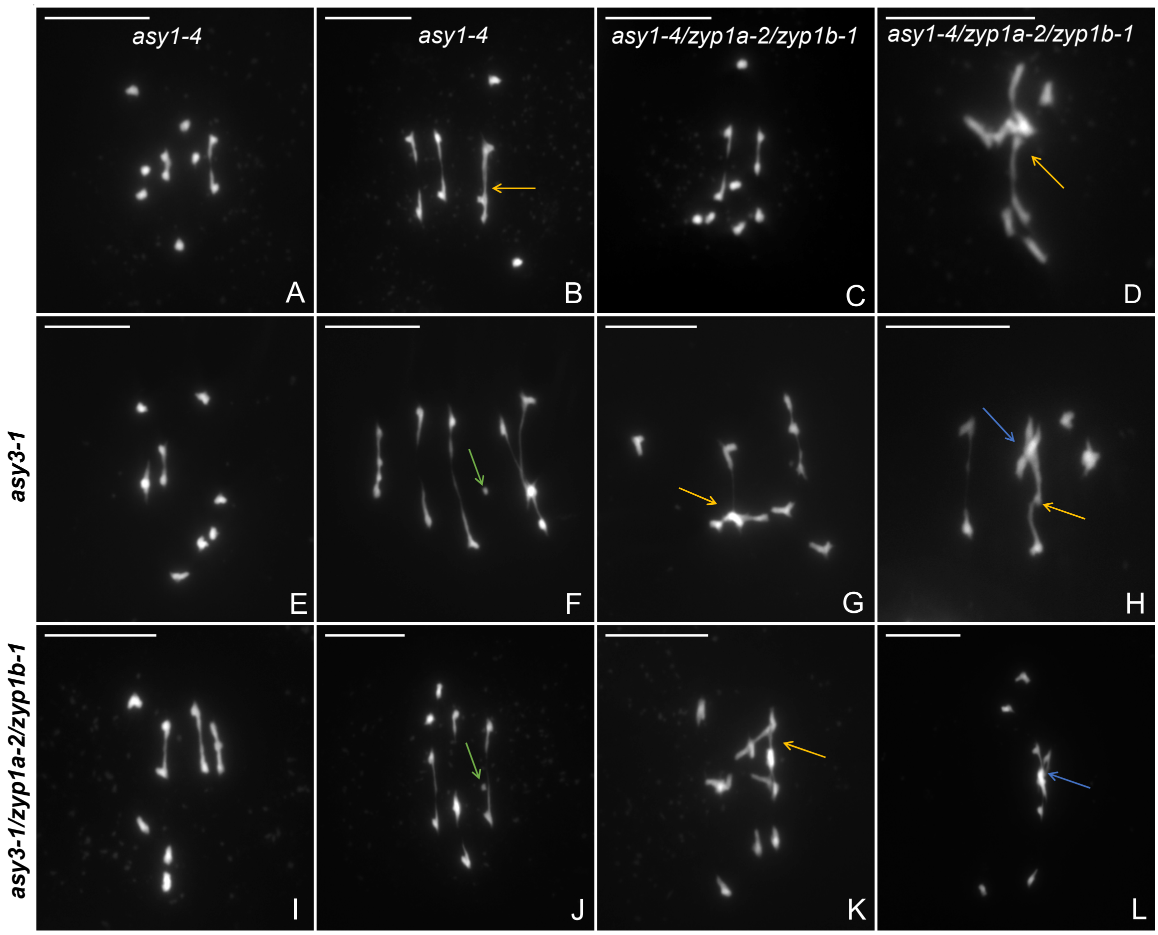

Fig. S5. Metaphase I defects in *zyp1 axis* double mutants. DAPI stained metaphase I chromosome spreads for *asy1-1* (*A-B*), *asy1-1/zyp1a-2/zyp1b-1* (*C-D*), *asy3-1* (*E-H*) and *asy3-1/zyp1a-2/zyp1b-1*. A representative image is shown for each line (*A, C, E, I*). In addition, cells displaying particular abnormalities discussed in text are shown. Potential interlocked bivalents (blue arrows), multivalents (yellow arrows) and chromosome fragments (green arrows) are indicated. Scale bars = 10μm.

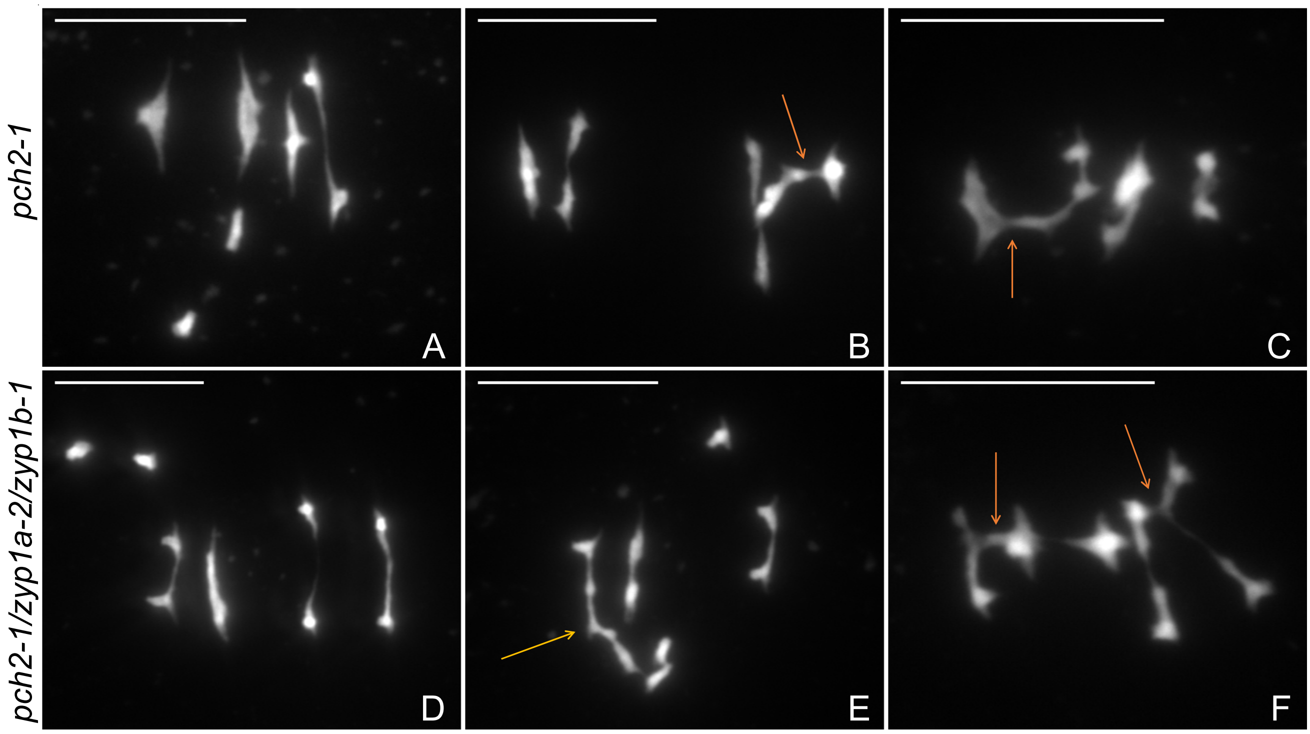

Fig. S6. Metaphase I defects in *pch2/zyp1* double mutants. DAPI stained metaphase I chromosome spreads for *pch2-1* (*A-C*) and *pch2-1/zyp1a-2/zyp1b-1* (*D-F*). A representative image is shown for both lines (*A, D*). In addition, cells displaying particular abnormalities discussed in text are shown. Aberrant connections between bivalents (orange arrows) and a potential multivalent (yellow arrow) are indicated. Scale bars = 10μm.

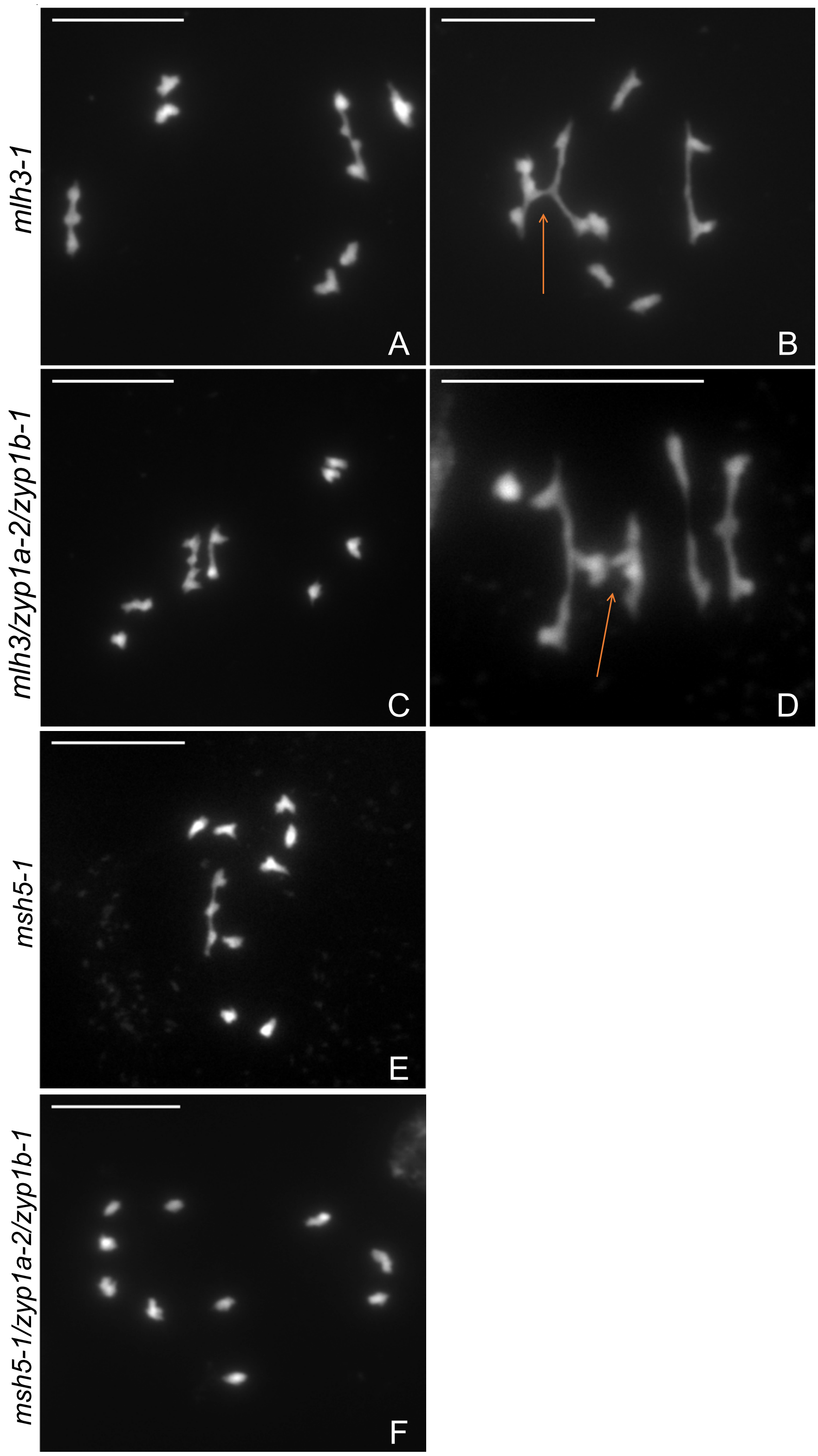

Fig. S7. Metaphase I defects in *mlh3/zyp1a-2/zyp1b-1* and *msh5-1/zyp1a-2/zyp1b-1* triple mutants. DAPI stained metaphase I chromosome spreads for *mlh3-1* (*A-B*), *mlh3/zyp1a-2/zyp1b-1* (*C-D*), *msh5-1* (*E*) and *msh5-1/zyp1a-2/zyp1b-1* (*F*). A representative image is shown for all lines (*A, C, E, F*). In addition, cells showing aberrant connections between bivalents (indicated by orange arrows) are shown. Scale bars = 10µm.

Table S1. Oligonucleotide sequences (5'-3') and primer pairs for genotyping mutants

ZYP1A gRNA	GAAGATGAGAAGCTTTGGAG
ZYP1B gRNA	GGATCGGCGAAGACGTACTT
zyp1_ns_1	CTCGCATTTGCTGGTTTAAAGAGTC
zyp1b_sp_1	TGCGTATATTGCTAGGTTTATATTG
salk_lb2	GTGCTTTACGGCACCTCGAC
zyp1a_sp_1	GAATAGTTAGCAGATTCATATTTCAC
zyp1a_sp_2	GCAATGAAAACGAGAAGCAGT
zyp1a_sp_3	TACCTGGTCGTTCCTCTGTG
zyp1a_sp_4	CAGAATCAGCGATTTCGC
LBA1	TGGTTCACGTAGTGGGCCATCG
zyp1b_sp_2	ACGATTTGAGAGAGGCGGTG
zyp1b_ sp_3	TACCTGGTCGTTCCTCTGTG
zyp1b_sp_4	TTGTAGCTTACTTTCTCGATCTCC
asy1_047272_F	ATGGTCCGATGATTGAGGAA
asy1_046272_R	TTTGCAAGCTGAACTCCTGA
MLH3_SALK_015849_F	TATGGCCTTCACAGCAACAG
MLH3_SALK_015849_R	TCCATAGCTTCTGGCCAATC
MSH5_SALK_110240_F	CATAAGTGCGCTCCTGCAC
MSH5_SALK_110240_R	TCACTAAGGCCTGCTGAATTTG
M13_F	GTAAAACGACGGCCAGT

Primer pairs:_ZYP1b T-DNA flanking PCR (zyp1_ns_1 & zyp1b_sp_1); ZYP1b LB PCR (salk_lb2 & zyp1b_sp_1); ZYP1a flanking PCR For *Hind*III digest (zyp1_ns_1 & zyp1a_sp_1); ZYP1a T-DNA flanking PCR (zyp1a_sp_2 & zyp1a_sp_3); ZYP1a LB PCR (zyp1a_sp_4 & LBA1); ZYP1b flanking PCR for 1bp Seq (zyp1b_sp_4 & zyp1b_sp_2); ASY1 T-DNA flanking PCR (asy1_047272_F & asy1_046272_R); ASY1 T-DNA LB PCR (asy1_047272_F & salk_lb2); ASY3 as in ; PCH2 as in); MLH3 T-DNA flanking PCR (MLH3_SALK_015849_F & MLH3_SALK_015849_R); MLH3 T-DNA LB PCR (salk_lb2 & MLH3_SALK_015849_R); MSH5 T-DNA flanking PCR (MSH5_SALK_110240_F & MSH5_SALK_110240_R); MSH5 T-DNA LB PCR (salk_lb2 & MSH5_SALK_110240_R).

Table S2. Quantification of aberrant structures at metaphase I in all genotypes

Genotype	n	Interlocked Chromosomes	Cells Containing Non-Homologous Connections	Multivalents	Cells Containing Fragments
WT	78	0	0	0	0
zyp1a-1	22	0	0	0	0
zyp1b-1	60	0	0	0	0
zyp1a-2/zyp1b-1	52	1	2	0	0
zyp1a-3/zyp1b-1	45	1	1	0	0
zyp1a-1/zyp1b-2	18	1	1	0	0
asy1-4	54	0	0	1	0
asy1-4/zyp1a-2/zyp1b-1	40	0	0	1	0
asy3-1	63	2	0	3	7
asy3-1/*zyp1a-2/zyp1b-1*	98	1	0	8	6
pch2-1	73	0	2	0	0
pch2-1/zyp1a-2/zyp1b-1	61	0	2	4	0
mlh3-1	67	0	2	0	0
mlh3-1/zyp1a-2/zyp1b-1	54	0	3	0	0
msh5-1	82	0	0	0	0
msh5-1/zyp1a-2/zyp1b-1	97	0	0	0	0

Table S3. Classes of fluorescent pollen scored in the I3bc intervals (A) and calculation of the interference ratios (B) for wild type and *zyp1a-3/zyp1b-1*.

A

Tetrad Class	WT	*zyp1a-3/zyp1b-1*
**A** (NCO)	1342	1480
**B** (I3b SCO)	755	913
**C** (I3c SCO)	217	262
**D** (I3b & I3c SCO)	7	43
**E** (I3b & I3c SCO)	10	54
**F** (I3b & I3c SCO)	4	41
**G** (I3b & I3c SCO)	7	35
**H** (I3b DCO)	6	45
**I** (I3c DCO)	1	2
**J** (I3b DCO & I3c SCO)	0	4
**K** (I3c DCO & I3b SCO)	0	1
**L** (13b DCO and I3C DCO)	0	0
Total	2349	2880

B

	WT	*zyp1a-3/zyp1b-1*
I3b ± SE<br>(p value WT vs *zyp1a-3/zyp1b-1*)	17.43 ± 0.57	23.98 ± 0.81<br>1.97×10^{-11}
I3c ± SE<br>(p value WT vs *zyp1a-3/zyp1b-1*)	5.34 ± 0.34	7.93 ± 0.38<br>1.73×10^{-7}
I3b without adjacent CO (cM)	18.81	24.26
I3b with adjacent CO (cM)	5.69	22.40
Interference Ratio I3b-I3c<br>(p value WT vs *zyp1a-3/zyp1b-1*)	0.3<br>± 0.055	0.92<br>± 0.079<br>4.62×10^{-11}

Table S4. Classes of fluorescent pollen scored in the I3bc intervals broken down for each of the three *zyp1a-3/zyp1b-1* mutant plants tested.

Tetrad Class	*zyp1a-3/zyp1b-1 U6*	*zyp1a-3/zyp1b-1 T1*	*zyp1a-3/zyp1b-1 I6*	Total
**A** (NCO)	461	497	522	1480
**B** (I3b SCO)	285	313	315	913
**C** (I3c SCO)	98	86	78	262
**D** (I3b & I3c SCO)	10	15	18	43
**E** (I3b & I3c SCO)	20	19	15	54
**F** (I3b & I3c SCO)	17	12	12	41
**G** (I3b & I3c SCO)	11	12	12	35
**H** (I3b DCO)	11	19	15	45
**I** (I3c DCO)	0	1	1	2
**J** (I3b DCO & I3c SCO)	0	1	3	4
**K** (I3c DCO & I3b SCO)	0	0	1	1
**L** (13b DCO and I3C DCO)	0	0	0	0
Total	913	975	992	2880

Table S5. Classes of fluorescent pollen scored in the I5b interval (A) and genetic distance (B).

A.

Tetrad Class	Tetrad Types	WT	*zyp1a-3/zyp1b-1*
**A** (NCO)	P	836	639
**B** (I5b SCO)	T	336	460
**C** (I5b DCO)	N	3	17
Total		1175	1116

B.

Genetic Distance (cM)	WT	*zyp1a-3/zyp1b-1*
15b ± SE<br>(p value WT vs *zyp1-2*)	15.06<br>± 0.78	25.18<br>± 1.26<br>4.22×10^{-12}

Table S6. Intra-interval analysis of I3b, I3c, I5b

Tetrad Class	I3b				I3C				I5b			
	WT		*zyp1a-3/zyp1b-1*		WT		*zyp1a-3/zyp1b-1*		WT		*zyp1a-3/zyp1b-1*	
	OBS	EXP	OBS	EXP	OBS	EXP	OBS	EXP	OBS	EXP	OBS	EXP
NCO	1560	1589.8	1744	1760.9	2103	2105.4	2438	2443.8	836	846.1	639	651.8
SCO	783	723.3	1087	1053.1	245	240.4	439	427.3	336	315.8	460	434.4
DCO	6	35.8	49	65.9	1	3.31	3	8.8	3	13.1	17	29.8
OBS/EXP DCO	0.17		0.74		0.30		0.34		0.23		0.57	
CHI.SQ	30.3		5.61		EXP < 5		4.18		9.23		7.24	
p	3.69×10^{-8}		0.018		NA		0.041		0.0024		0.0071	

Table S7. Analysis of the 420 interval in the fluorescent seed recombination assay.

WT

Plant	None (Proportion)	Red Only (Proportion)	Green Only (Proportion)	Both (Proportion)	Total	$\frac{\text{Red}}{\text{Green}}$	Map Distance (cM)
B3	224 (0.16)	125 (0.09)	106 (0.08)	867 (0.66)	1322	1.18	19.34
D6	198 (0.15)	146 (0.11)	133 (0.10)	878 (0.65)	1355	1.10	23.31
P6	200 (0.16)	107 (0.08)	127 (0.10)	845 (0.66)	1279	0.84	20.37
W6	190 (0.15)	108 (0.08)	110 (0.09)	872 (0.68)	1280	0.98	18.80
X5	244 (0.15)	148 (0.09)	170 (0.10)	1096 (0.66)	1658	0.87	21.49
Total	1056 (0.15)	634 (0.09)	646 (0.09)	4558 (0.66)	6894	0.98	20.66

zyp1a-2/zyp1b-1

Plant	None (Proportion)	Red Only (Proportion)	Green Only (Proportion)	Both (Proportion)	Total	$\frac{\text{Red}}{\text{Green}}$	Map Distance (cM)
A6	175 (0.10)	247 (0.15)	209 (0.12)	1069 (0.63)	1700	1.18	31.92
E5	201 (0.11)	292 (0.16)	206 (0.11)	1127 (0.62)	1826	1.42	32.58
G1	232 (0.10)	303 (0.14)	293 (0.13)	1395 (0.63)	2223	1.03	31.90
P4	170 (0.12)	190 (0.13)	191 (0.13)	886 (0.62)	1437	0.99	31.46
R2	212 (0.12)	232 (0.13)	223 (0.13)	1077 (0.62)	1744	1.04	30.85
U3	184 (0.11)	206 (0.12)	217 (0.13)	1065 (0.64)	1672	0.95	29.71
Total	1174 (0.11)	1470 (0.14)	1339 (0.13)	6619 (0.62)	10602	1.10	31.40

References

1. S. J. Clough, A. F. Bent, Floral dip: a simplified method for Agrobacterium-mediated transformation of Arabidopsis thaliana. *Plant J* **16**, 735-743 (1998).
2. J. D. Higgins, E. Sanchez-Moran, S. J. Armstrong, G. H. Jones, F. C. Franklin, The Arabidopsis synaptonemal complex protein ZYP1 is required for chromosome synapsis and normal fidelity of crossing over. *Genes Dev* **19**, 2488-2500 (2005).
3. E. Sanchez-Moran, S. J. Armstrong, J. L. Santos, F. C. Franklin, G. H. Jones, Variation in chiasma frequency among eight accessions of Arabidopsis thaliana. *Genetics* **162**, 1415-1422 (2002).
4. L. E. Berchowitz, G. P. Copenhaver, Fluorescent Arabidopsis tetrads: a visual assay for quickly developing large crossover and crossover interference data sets. *Nat Protoc* **3**, 41-50 (2008).
5. R. Peterson, J. P. Slovin, C. Chen, A simplified method for differential staining of aborted and non-aborted pollen grains. *International Journal of Plant Biology* **1**, e13 (2010).
6. C. Melamed-Bessudo, E. Yehuda, A. R. Stuitje, A. A. Levy, A new seed-based assay for meiotic recombination in Arabidopsis thaliana. *Plant J* **43**, 458-466 (2005).
7. P. A. Ziolkowski *et al.*, Juxtaposition of heterozygous and homozygous regions causes reciprocal crossover remodelling via interference during Arabidopsis meiosis. *Elife* **4** (2015).
